# Taxonomy of the Genus *Porella* (Porellaceae, Marchantiophyta) on the Korean Peninsula

**DOI:** 10.3390/plants14081260

**Published:** 2025-04-21

**Authors:** Hyun Min Bum, Seung Jin Park, Narae Yun, Vadim A. Bakalin, Seung Se Choi

**Affiliations:** 1 Department of Life Science, Jeonbuk National University, Jeonju 54896, Republic of Korea; know8318@jbnu.ac.kr; 2 Division of Botany, Honam National Institute of Biological Resources, Mokposi 58762, Republic of Korea; moss89@hnibr.re.kr (S.J.P.); narae@hnibr.re.kr (N.Y.); 3 Botanical Garden-Institute, Far Eastern Branch of the Russian Academy of Sciences, Makovskogo Street, 142, Vladivostok 690024, Russia; 4 Team of National Ecosystem Survey, National Institute of Ecology, Seocheon 33657, Republic of Korea

**Keywords:** Korean Peninsula, Marchantiophyta, *Porella*, Porellaceae, phylogeny

## Abstract

This paper provides a revision of *Porella* in the Korean Peninsula based mostly on a study of the collections housed in the herbaria of Jeonbuk National University (JNU) using an integrative approach to systematize the liverwort, as well as a study of the types of several species and available literature sources. In total, 17 species were recorded, including six taxa (*Porella acutifolia* ssp. *tosana, P. platyphylla*, *P. perrottetiana*, *P. pinnata*, *P. spinulosa*, and *P. subobtusa*) whose identities were not confirmed with the available materials and were suspected to be recorded by mistake. Two species are described as new to science. *Porella koreana* sp. *nov*. is morphologically similar to *P. caespitans* and *P. densifolia*; however it has an acute apex, and cells in the middle of the dorsal lobe have convex trigones. *Porella chulii* sp. nov. is somewhat morphologically similar to *P. japonica*; however, it has a dorsal leaf lobe margin that is slightly incurved, and the cells in the middle of the dorsal lobe are 20–25 μm in size. Each confirmed species is annotated by morphological descriptions based on available Korean material, data on ecology, its distribution, specimens examined, and illustrations. The identification key for *Porella* taxa known in Korea is provided.

## 1. Introduction

The genus *Porella* L. is a representative genus that is known to be difficult to classify morphologically. Although there are many regional revisions of this genus, its general evolution and phylogenetic estimations are rather rare [1,2]. The family Poreallaeae Cavers is a relatively large group of bryophytes that includes monotypic *Ascidiota* C. Massal. and polytypic *Porella*. The genus *Porella* comprises 120 taxa in the genus *Porella* (85 species, 7 subspecies, and 28 varieties), with 21 of undoubted status (three asterisks), 92 taxa having a knowledge problem (following the terminology accepted in Söderström et al. [3], two asterisks), and 7 taxa with serious doubts about their status (one asterisk in l.c.) [3]. For a long time, *Macvicaria* was deeply nested within *Porella* and synonymized with the latter [2]. Porellaceae is considered as the most basal taxon among the Class Jungermanniopsida of the Phylum Marchantiophyta (liverworts), and its main distinguishing characteristics are leaves divided into dorsal lobes and ventral lobules, large regular underleaves, and sporophytes enclosed by a shoot calyptra and perianth [4].

*Porella* species are most diverse in tropical regions and very rarely in boreal and arctic regions. Approximately 120 taxa are distributed worldwide, and approximately 60 taxa are distributed in East Asia, including 51 taxa in China, 19 taxa in Japan, 11 taxa in Far East Russia, and 17 recorded taxa on the Korean Peninsula [5,6,7,8,9,10,11].

The morphological features of leaves and underleaves, including their decurrencies along the stem, are the most valuable features considered in *Porella* identification [5]. It is not easy to determine the limits of each taxon because the ranges of variation in these major distinguishing traits are very large or not clear. Taxa that have been described or studied were identified without considering the ranges of variation in these overall taxa.

Therefore, the aim of this study was to establish the taxonomic position of each taxon by examining the morphological characteristics and molecular phylogenetic characteristics of the genus *Porella* on the Korean Peninsula. In addition, we tried to reveal the taxonomic identities of taxa that have been recorded for the Korean Peninsula but were never recollected.

### Historical Background Wordwide and of the Korean Peninsula

The genus *Porella* is one of the oldest recognized genera of liverworts. It was originally described in *Species Plantarum* by Carl von Linne in 1753 with the species *P. pinnata* L. [12]. In 1822, Dumortier did not recognize the genus *Porella* and described the genus *Madotheca* Dumort. based on its perianth features [13]. After that, *Jungermannia platyphylla* L., *J. laevigata* Schrad., and *J. thuja* Dicks. were subsequently transferred to *Madotheca platyphylla* (L.) Dumort., *M. laevigata* (Schrad.) Dumort., and *M. thuja* (Dicks.) Dumort. [13]. Later, Pfeiffer recognized Carl von Linne’s *Porella* in 1855, and *M. platyphylla* (=*P. platyphylla*), *M. laevigata* (=*P. laevigata*), and *M. rivularis* Nees (=*P. rivularis* (Nees) Pfeiff.) were transferred to the genus *Porella* [14]. Many taxa of *Madotheca* in East Asia were transferred to the genus *Porella* by Hattori [15,16,17,18,19,20,21,22]. Currently, the genus *Madotheca* and all taxa published within the genus have been transferred to the genus *Porella*, and the genus *Madotheca* is treated as a synonym for the genus *Porella* [3]. Additionally, Porellaceae species with highly crispate leaves were described as a new genus, *Macvicaria* [23]. *Porella ulophylla*, which has severely crispate leaves, was first described as a new species, *Madotheca ulophylla* [24], and was then transferred to *Porella ulophylla* [25] and then to *Mavicaria ulophylla* [16]. However, molecular phylogenetic studies have shown that *Mavicaria* is included in the genus *Porella*; therefore, the transfer of the latter to *Porella* is now accepted [2].

The first record of this genus on the Korean Peninsula was by Stephani [26], who recorded Korea as the area of distribution of *Madotheca tosana* Steph. Currently, *M. tosana* on the Korean Peninsula is considered a misidentified *P. oblongifolia* based on the leaf marginal teeth characteristics [11].

Uno and Takahasi [27] recorded *M. setigera* (=*P. caespitans*) in a study of bryophytes on Mt. Jiri. Ando [28] reported three taxa for Korea, *P. vernicosa*, *P. grandiloba*, and *P. gracillima*, according to the leaf type (dorsal leaf lobe, ventral leaf lobe, and underleaves) in a study on the complex of *Porella*. Hong and Kim [29] reported six previously unrecorded species for the Korean Peninsula: *P. vernicosa* ssp. *fauriei* (=*P. fauriei*), *P. grandiloba*, *P. japonica*, *P. setigera* (=*P. caespitans*), *P. ulophylla*, and *P. vernicosa*.

Most of the *Porella* taxa on the Korean Peninsula were recorded as part of regional bryophyte studies. In North Korea, Kim et al. [30] reported 15 taxa among bryophytes on Mt. Geumgang. Hong [31] studied the regional flora of the Korean Peninsula and reported that 11 taxa were distributed on the Korean Peninsula. Choe [6], in an investigation of bryophytes in Korea, recorded 12 taxa. The most recent liverwort checklist for the peninsula includes data on the distribution of 16 *Porella* species [11].

As such, the study of the genus *Porella* on the Korean Peninsula has been performed as part of the bryophyte flora; however, there has been no taxonomic study on the entire taxon, only a list of major mountain areas.

## 2. Results

A total of 13 taxa, including 11 taxa and 2 new-for-science taxa, were selected for the genus *Porella* on the Korean Peninsula. Six taxa, such as those with suspected distributions, were studied.

The length of the *trn*L-F region analyzed in this study for the genus *Porella* was confirmed to be 588 bp, with a G+C content of 31.8%. The rate of nucleotide variation in the *trn*L-F region among the taxonomic groups within the genus *Porella* was 14.6%. The *trn*L-F alignment resulted in a total of 588 bp, with 86 bp identified as variable sites and 60 bp identified as phylogenetically informative sites. The length of the resulting phylogenetic tree was 110, and 110 phylogenetic trees were generated with a consistency index (CI) of 0.88 [32] and a retention index (RI) of 0.96 [33].

Among these phylogenetic trees, the 50% majority-rule consensus tree was selected, and bootstrap analysis was performed, with the bootstrap values recorded on the phylogenetic tree. Based on the nucleotide sequences of the *trn*L-F region, 67 individuals from 19 taxa of the genus *Porella*, along with 1 taxon from the genus *Ascidiota* as an outgroup, formed a monophyletic group with a high bootstrap value of 100%. Notably, *P. chulii* and *P. koreana* were clearly recognized as distinct, independent species within the phylogenetic tree. *P. chulii* was identified as a separate taxon from its closely related species *P. japonica*, whereas *P. koreana* was confirmed as an independent taxon distinct from the *P. caespitans* complex group.

**Figure 1:**
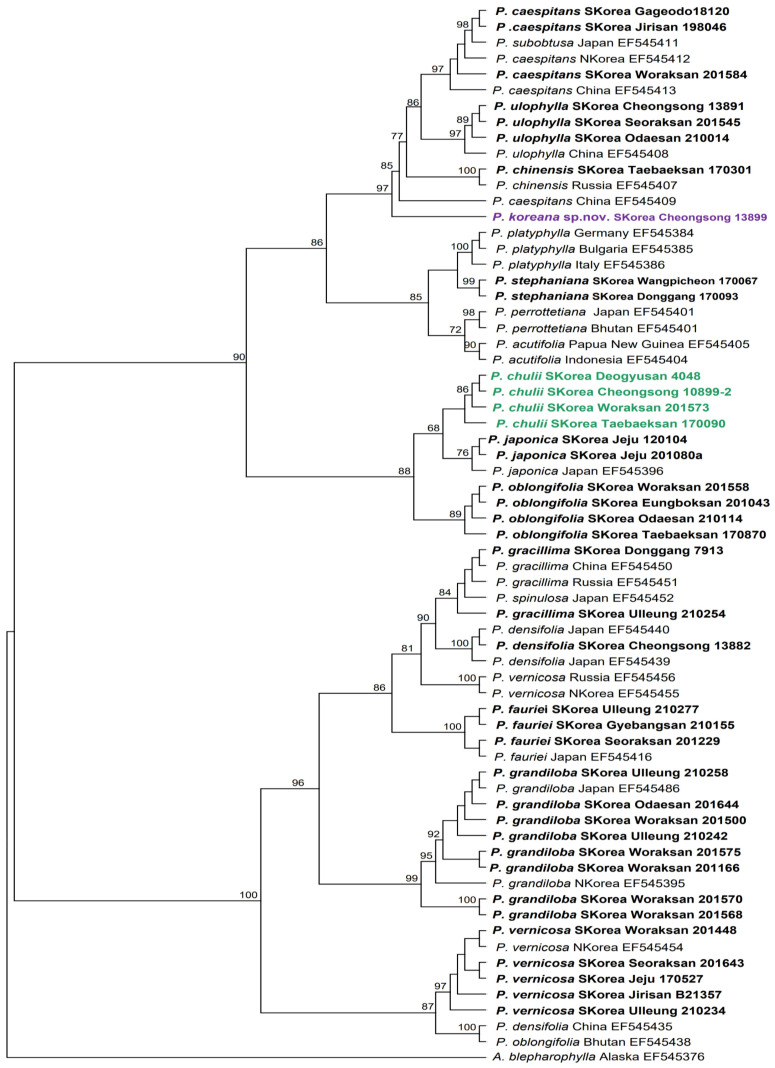
Maximum likelihood tree inferred from *trn*L-F chloroplast gene sequences, illustrating the evolutionary relationships among the analyzed species. Korean taxa are represented in bold, while *P. chulii* is shown in green, and *P. koreana* is shown in purple.

The cladistic resolution of the phylogenetic tree was determined to be 89.4%, based on a total of 38 internal nodes, 34 of which (≥80% bootstrap value) were fully resolved. This finding indicates a well-supported and highly resolved phylogenetic structure (Figure 1).

## 3. Taxonomic Treatment of *Porella* L. on the Korean Peninsula

***Porella* L., Sp. Pl. 1: 1106, 1753.**

Type Species: *Porella pinnata* L., Sp. Pl. 1: 1106, 1753. (North America, Pennsylvania).

Description: Plants prostrate to ascending, yellowish green, green, brownish green, and yellowish brown. Stems regularly pinnately or bipinnately branched, cross section, cortex cells thick-walled in 1–5 layers, and inner cells thin-walled. Rhizoids sparse on underleaves. Leaves divided larger on dorsal lobes and smaller on ventral lobes. Dorsal leaf lobes: imbricate, obliquely triangular ovate, ovate, elliptical, apex acuminate, acute, obtuse, rounded, and with entire or tooth margin. Ventral leaf lobes obliquely spreading, oblong ovate, oblong, narrows elliptic, decurrent for short or long of stem width, apex acute to obtuse or rounded, and entire margin or with teeth. Underleaves oblong, obovate, ovate, elliptical, short or long decurrent for stem width on both sides, apex acute, obtuse to rounded, and with entire margin variously toothed. Cells in dorsal lobe middle thin walled, slightly thickened, and trigones concave, with moderate triangular or convex shapes; cuticle smooth. Oil bodies 10–40 per cells, spherical to elliptic, and homogenous. Dioicous. Androecia: terminal or lateral on branches and uniandrous. Gynoecia terminal on lateral branches; perianth strongly or weakly dorsiventrally compressed. Seta massive and short. Capsules emergent from the perianth or fully immersed. Spores multicellular at maturity and papillose. Specialized organs of vegetative reproduction are not known.

There are approximately 120 taxa of the genus *Porella* distributed in the temperate and subtropical regions of the Northern Hemisphere and Eurasia, including North America [3]. Among them, East Asia is the region with the highest diversity, with approximately 60 taxa distributed [1,5]. As mentioned in the previous research history, 17 taxa were recorded as distributed on the Korean Peninsula; however, taxonomic treatment was performed on a total of 13 taxa, including 11 taxa and 2 new-for-science taxa, excluding 6 taxa that were dubiously reported. The differences between the 13 taxa, including newly described *P. koreana* and *P. chulii* have been compiled and are outlined in Table 1.

Key to *Porella* taxa on the Korean Peninsula.

1. Dorsal leaf lobe with a prominently acuminate to acute apex……………………... 2.

1. Dorsal leaf lobe with rounded to obtuse, rarely acute apex…………………….…. 4.

2. Dorsal leaf lobe with prominently acuminate apex, cells in dorsal lobe middle triangular trigones …..................................................................................... 1. *P. caespitans*

2. Dorsal leaf lobe with acute apex, cells in dorsal lobe middle convex trigones…. 3.

3. Dorsal leaf lobe entire, habitat in wind holes………………………… 12. *P. koreana*

3. Dorsal leaf lobe dentate, habitat on calcareous rocks……………… 9. *P. stephanina*

4. Dorsal leaf lobe dentate to variously ciliate……………………………………….... 5.

4. Dorsal leaf lobe entire …………………………………………………………..…….. 9.

5. Dorsal leaf lobe densely toothed, strongly incurved to dorsal side apex………… 6.

5. Dorsal leaf lobe with sparsely toothed apex, slightly incurved to flat apex……... 7.

6. Underleaves nearly triangular with truncate apex not or sparsely dentate ………………………………………………………………………………… 4. *P. fauriei*

6. Underleaves lingulate to obovate, with apex densely dentate to shortly ciliate …………………………………………………………………………….. 11. *P. vernicosa*

7. Underleaves decurrent for 0.8–1.2 of stem width ............................... 8. *P. oblongifolia*

7. Underleaves decurrent for 0.1–0.3 of stem width .......................................................8.

8. Dorsal leaf lobe margin plane, cells in dorsal lobe middle 25–30 μm ................................................................................................................ 7. *P. japonica*

8. Dorsal leaf lobe margin slightly incurved, cells in dorsal lobe middle 20–25 μm ................................................................................................................... 13. *P. chulii*

9. Ventral leaf lobe decurrent for 0.1–0.5 of stem width................................................ 10.

9. Ventral leaf lobe decurrent for 0.5–1.0 of stem width............................................... 11.

10. Underleaves decurrent for 0.1–0.2 of stem width, ventral leaf lobe 0.2–0.3 mm width....................................................................................................... 6. *P. grandiloba*

10. Underleaves decurrent for 0.5–0.6 of stem width, ventral leaf lobe 0.3–0.6 mm width......................................................................................................... 5. *P. densifolia*

11. IKI (potassium iodide and iodine) reaction positive............................................. (*Porella platyphylla* the discussions on distribution, description and illustrations are in Bakalin and Klimova [10])

11. IKI reaction negative................................................................................................... 12.

12. Dorsal leaf lobe plane at base, cells in dorsal lobe middle 15–20 μm. 6. *P. gracillima*

12. Dorsal leaf lobe with undulate at base, cells in dorsal lobe middle 30–40 μm. 13.

13. Dorsal leaf lobe with undulate at base..................................................... 2. *P. chinensis*

13. Dorsal leaf lobe with undulate entire..................................................... 10. *P. ulophylla*

(1) ***Porella caespitans*** (Steph.) S. Hatt., J. Hattori Bot. Lab. 33: 50, 1970 (Figure 2).

Basionym.: *Madotheca caespitans* Steph., Mem. Soc. Nat. Sci. Nat. Math. Cherbourg 29: 218, 1894.

*Porella setigera* (Steph.) S. Hatt., J. Jap. Bot, 20: 107, 1944.; *Porella caespitans* var. *setigera* (Steph.) S. Hatt., J. Hattori Bot. Lab. 33: 53, 1970.; *Porella caespitans* var. *cordifolia* (Steph.) S. Hatt., Bryol. Res. 10(5): 133. 2011.

Description. Plants prostrate to ascending, yellowish green, green, and browinsh green, 40.0–60.0 mm long and 2.5–3.8 mm wide. Stem regularly pinnately or bipinnately branched, cross section 0.5–0.6 × 0.35–0.4 mm, cortex cells thick-walled in 2–3 layers, 15.0–25.0 × 7.5–10.0 μm, inner cells 25.0–32.5 × 20.0–25.0 μm. Rhizoids sparse. Dorsal leaf lobes imbricate, obliquely triangular ovate, 2.2–2.5 × 1.1–1.4 mm, dorsally arcuately inserted and barely decurrent, long acuminate apex, with entire margin, rarely 1–2 teeth. Ventral leaf lobe obliquely spreading, oblong, oblong triangular, oblong ovate, 0.6–0.87 × 0.3–0.5 mm, decurrent for 1.0–1.2 of stem width, apex acute to obtuse or shortly bilobed, with 2–3 teeth. Underleaves sheathing the stem near base, decurrent for 1.0–1.2 of stem width on both sides, ovate to triangular ovate, 0.6–1.0 × 0.6–0.75 mm, apex acute to obtuse, commonly bilobed near apex, with with entire to 1–2 teeth. Cells in dorsal lobe middle subisodiametric to oblong, 30.0–37.5 × 25–30 μm, thin walled, trigones moderate triangular, near apex 20.0–27.5 × 20.0–27.5 μm, near base 37.5–50.0 × 30.0–37.5 μm, trigones triangular convex; cuticle smooth. Oil bodies 15–30 per cells, spherical to elliptic, homogenous, 2.5–5.0× 2.5–3.0 μm. (Androecia terminal on branches of the first or second order, with 1-several pairs of sterile leaves between branch origin and antheridial bracts, spicate, with 3–8 pairs of bracts, monoandrous [10]).

Distribution: Japan, China, the Russian Far East, Bhutan, and India [5]. In the Korean Peninsula, it is found on Jeju-do, Jellaman-do, Jellabuk-do, Chungcheongnam-do, Chungcheongbuk-do, Gyeongsangnam-do, Gyeongsangbuk-do, Gyeonggi-do, Gangwon-do, Pyeonganbuk-do, Jagang-do, Hamgyeongnam-do, and Hamgyeongbuk-do [6,8,11,31].

Comment: This species was described by Stephani [34] as a new species, *Madotheca caespitans*, based on specimens collected by S.M. Delavay in Yunnan, China [34]. Later, Hattori [19] studied the holotype specimen and transferred it to the genus *Porella*. Depending on the shape of the leaf variation, it is sometimes classified into several varieties and subspecies [19]; however, in this study, it was treated according to the view of Konstantinova et al. [35], in which it is regarded as a taxon.

**Figure 3:**
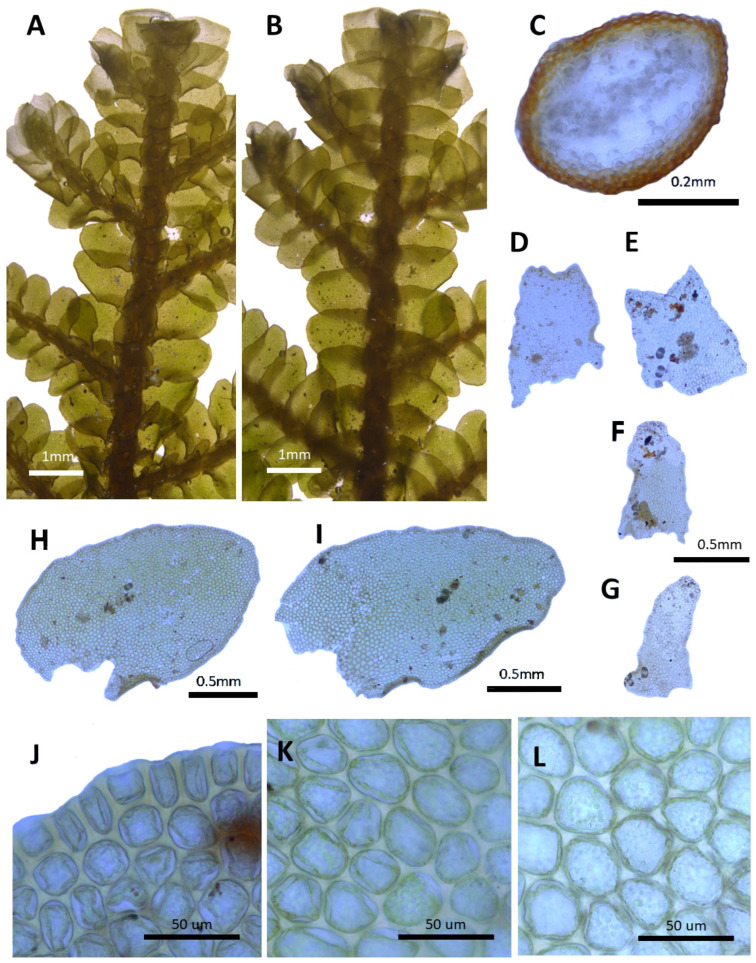
*Porella chinensis* (Steph.) S. Hatt.: (**A**) plant habit, ventral view; (**B**) plant habit, dorsal view; (**C**) cross section of the stem; (**D**,**E**) underleaves; (**F**,**G**) ventral lobes; (**H**,**I**) dorsal lobes; (**J**) marginal cells of the dorsal lobe; (**K**) middle cells of the dorsal lobe; (**L**) basal cells of the dorsal lobe. All images from Bum and S.S. Choi, 170301 (JNU).

(2) ***Porella chinensis*** (Steph.) S. Hatt., J. Hattori Bot. Lab. 30: 131, 1967 (Figure 3).

Basionym: *Madotheca chinensis* Steph., Mém. Soc. Nat. Sci. Nat. Math. Cherbourg 29: 218, 1894.

Description: Plants prostrate to ascending, brownish green, green, and yellowish green, with dimensions of 40.0–60.0 mm long and 2.5–3.1 mm wide. Stems irregularly pinnately branched; a cross section of 0.4–0.5 × 0.33–0.36 mm; thick-walled cortex cells in 3–5 layers, with dimensions of 10.0–12.5 × 7.5–10.0 μm; and thin-walled inner cells with dimensions of 20.0–27.5 × 20.0–27.5 μm. Rhizoids sparse or rarely few at underleaf bases. Dorsal leaf lobes imbricate, obliquely elliptical, elliptical ovate, with dimensions of 1.6–2.0 × 1.0–1.3 mm, dorsally arcuately inserted and slightly decurrent, rounded apex, and with entire margin and slightly undulate. Ventral leaf lobes obliquely spreading, elliptical, narrowly elliptical, with dimensions of 0.6–0.8 × 0.3–0.4 mm, decurrent for 1.0–1.2 of the stem width, and apex acute to obtuse with entire. Underleaves sheathing the stem near the base, decurrent for 1.0–1.5 of the stem width on both sides, oblong, with dimensions of 0.5–0.7 × 0.4–0.5 mm, and apex obtuse to truncate with entire margin. Cells in the middle dorsal lobe subisodiametric to oblong, with dimensions of 30.0–37.5 × 25.0–30.0 μm, thin walled, trigones moderate triangular to convex; thick-walled near the apex, with dimension of 12.5–17.5 × 12.5–17.5 μm; and near base with dimensions of 32.5–40.0 × 20.0–27.5 μm; trigones triangular to convex; cuticle smooth. Oil bodies 15–30 per cells, spherical to elliptic, homogenous, and with dimensions of 2.5–5.0 × 2.5–3.0 μm. (Androecia terminal on branches of the first or second order, with 0–1 pairs of sterile leaves between branch origin and antheridial bracts, spicate, with 5–12 pairs of bracts [10].)

Habitat: This species grows on the shady rocks in mountainous areas.

Distribution: China (Yunnan, Sichuan, Shaanxi, and Hunan), India (Himalaya), and Russia (Siberia, Amur) [5]. In the Korean Peninsula, it is found in Taebaeksan and Deokhangsan.

Comment: This species was reported as an unrecorded species in Korea based on specimens collected from Deokhangsan by Choi et al. [36]. Although it shares similarities with *P. grandiloba* due to the rounded or obtuse apex of the dorsal lobes, it differs in having underleaves and ventral lobes that decurrent down along the stem.

**Figure 4:**
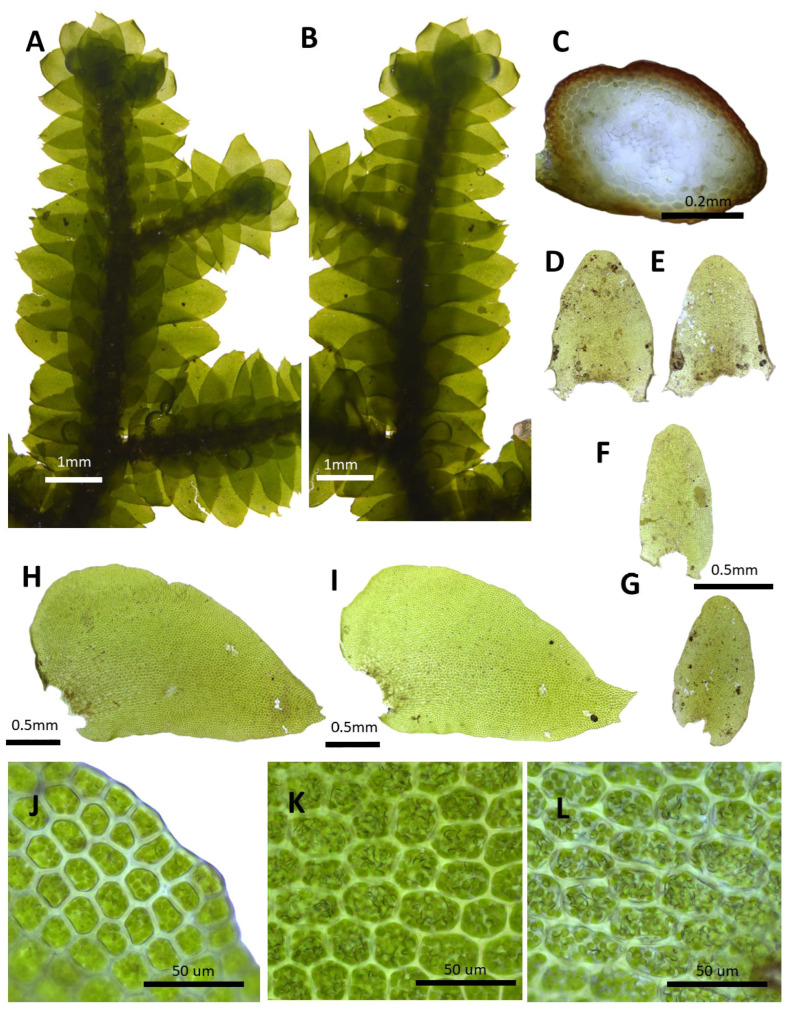
*Porella densifolia* (Steph.) S. Hatt.: (**A**) plant habit, ventral view; (**B**) plant habit, dorsal view; (**C**) cross section of the stem; (**D**,**E**) underleaves; (**F**,**G**) ventral lobes; (**H**,**I**) dorsal lobes; (**J**) marginal cells of the dorsal lobe; (**K**) middle cells of the dorsal lobe; (**L**) basal cells of the dorsal lobe. All images from Bum and S.S. Choi, B22349 (JNU).

(3) ***Porella densifolia*** (Steph.) S. Hatt., J. Jap. Bot. 20: 109. 1944 (Figure 4).

Basionym: *Madotheca densifolia* Steph., Mém. Soc. Nat. Sci. Nat. Math. Cherbourg 29: 219, 1894.

Description: Plants prostrate to ascending, yellowish green, green, and brownish green, with dimensions of 30.0–50.0 mm long and 3.0–3.75 mm wide. Stems irregularly pinnately branched; a cross section of 0.45–0.5 × 0.30–0.32 mm; thick-walled cortex cells in 3–4 layers, with dimensions of 7.0–10.0 × 5.0–7.5 μm; and thin-walled inner cells with dimensions of 20.0–25.0 × 20.0–25.0 μm. Rhizoids sparse or rarely few at underleaf bases. Dorsal leaf lobes imbricate, obliquely triangular ovate, elliptical ovate, ovate, with dimensions of 2.2–2.7 × 1.1–1.4 mm, dorsally arcuately inserted and slightly decurrent, apex acute, rarely obtuse, with two teeth, with entire margin, and slightely undurate. Ventral leaf lobes obliquely spreading, elliptical ovate, elliptical, with dimensions of 0.6–1.0 × 0.3–0.6 mm, decurrent for 0.1–0.4 of the stem width, apex rounded, obtuse, and with entire margin. Underleaves sheathing the stem near the base, slightly decurrent for 0.5–0.6 of the stem width on both sides, elliptical ovate, elliptical, with dimensions of 0.6–1.0 × 0.5–0.6 mm, apex rounded, obtuse, 2–3 teeth on the base of both sides, and with entire margin. Cells in the middle dorsal lobe subisodiametric to oblong, with dimensions of 30.0–37.5 × 17.5–25.0 μm, thin walled, trigones concave, small triangular; thick-walled near the apex, with dimensions of 12.5–17.5 × 12.5–17.5 μm; and dimensions of 32.5–37.5 × 22.5–27.5 μm near the base, trigones triangular to convex; cuticle smooth. Oil bodies 20–30 per cells, spherical to elliptic, homogenous, and with dimensions of 4.0–5.0 × 2.0–3.0 μm. Gynoecia not seen, as described and illustrated by Hara [37].

Habitat: This species grows on moist and shady rocky areas.

Distribution: Japan, China, and Taiwan [5]. In the Korean Peninsula, it is found in Chilbosan, Ogasan, Seonggan, Sinyang, Cheongsong, and Mudengsan [6,8,11,31].

Comment: This species was first described as a new species, *Madotheca densifolia*, by Stephani [34] based on specimens collected by M.S. You in China. Hattori [15] subsequently transferred it to the genus *Porella*. This species is distinguished from related taxa within the genus by its unique characteristic of having ventral lobes longer than the length of the underleaves, as well as its elliptical shape. Compared with this taxon, *P. densifolia* var. *oviloba* (Steph.) N. Kitag., which is distributed in Japan, does not show significant differences in underleaves and cell size. However, due to the inability to confirm the type specimen and other general specimens, further conclusions were deferred.

**Figure 5:**
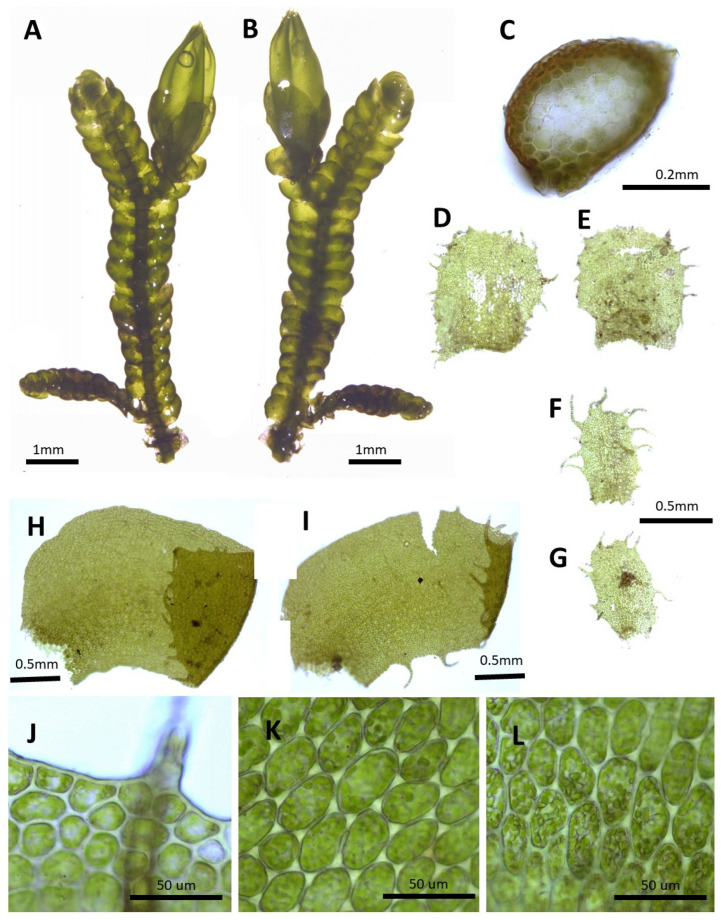
*Porella fauriei* (Steph.) S. Hatt.: (**A**) plant habit, ventral view; (**B**) plant habit, dorsal view; (**C**) cross section of the stem; (**D**,**E**) underleaves; (**F**,**G**) ventral lobes; (**H**,**I**) dorsal lobes; (**J**) marginal cells of the dorsal lobe; (**K**) middle cells of the dorsal lobe; (**L**) basal cells of the dorsal lobe. All from Bum and S.S. Choi, 210155 (JNU).

(4) ***Porella fauriei*** (Steph.) S. Hatt., J. Jap. Bot. 20(2): 109. 1944 (Figure 5).

Basionym: *Madotheca faurieri* Steph., Sp. Hepat. (Stephani) 4: 315, 1910

Description: Plants prostrate to ascending, yellowish green, green, and brownish green, with dimensions of 20.0–30.0 mm long and 1.2–1.5 mm wide. Stems irregularly pinnately branched; with a cross section of 0.30–0.35 × 0.24–0.26 mm; thick-walled cortex cells in 1–2 layers, with dimensions of 10.0–15.0 × 10.0–12.5 μm; and thin-walled inner cells, with dimensions of 25.0–30.0 × 25.0–30.0 μm. Rhizoids sparse or rarely few at underleaf bases. Dorsal leaf lobes imbricate, obliquely oblong, elloptical, elliptical ovate, strongly incurved to dorsal side 1/3–1/2 part, arcuately inserted, with dimensions of 1.0–1.2 × 0.7–0.9 mm, dorsally arcuately inserted and not decurrent, apex rounded, obtuse, crispate to entire, with 10–20 cilia, cilia longer near the apex, and ventral margin sparsely toothed to entire. Ventral leaf lobes obliquely spreading, lingulate to obliquely obovate, with dimensions of 0.3–0.5 × 0.2–0.4 mm, not decurrent, apex rounded, obtuse, with 10–15 teeth, and densely to sparsely toothed. Underleaves sheathing the stem near the base, shortly decurrent for 0.2–0.3 of the stem width on both sides, obovate, orbicular, deflexed, with dimensions of 0.6–0.7 × 0.5–0.65 mm, apex rounded, obtuse, with teeth, and teeth in the middle of the lateral side. Cells in the middle dorsal lobe subisodiametric to oblong, with dimensions of 20.0–27.5 × 17.5–22.5 μm, thin walled, trigones triangular slightly convex; thin-walled near the apex, with dimensions of 12.5–17.5 × 10.0–12.5 μm; and with dimensions of 30.0–37.5 × 17.5–22.5 μm near the base; cuticle smooth. Oil bodies 15–30 per cells, spherical to elliptic, homogenous, and with dimensions of 2.5–3.5 × 2.0–3.0 μm. Dioicous. Gynoecia on short lateral braches, perianth with dimensions of 3.0–3.2 × 1.2–1.5 mm, eliiptical ovate, and with keels.

Habitat: This species grows on shady and relatively dry areas on mountain slopes.

Distribution: Japan, China, and the Russian Far East. In the Korean Peninsula, it is found in Gwanmobong, Rimyeongsu, Chailbong (Bujeon), Ungeosusan, Chilbosan, Seonggan, Myohyangsan, Biraebong, Chuaesan, Heulyeongsan, Geumgangsan, Seoraksan, Odaesan, Gyebangsan, Ulleungdo, Jirisan, and Hallasan [6,8,11,31].

Comment: This species was first described as a new species, *Madotheca fauriei*, by Stephani [26] based on specimens collected in Kanita, Japan. Later, Hattori [15] transferred it to the genus *Porella*. This species is similar to *P. vernicosa* in having serrations on the dorsal leaf lobes and curved tips, but it is distinguished by its lingulate underleaves.

**Figure 6:**
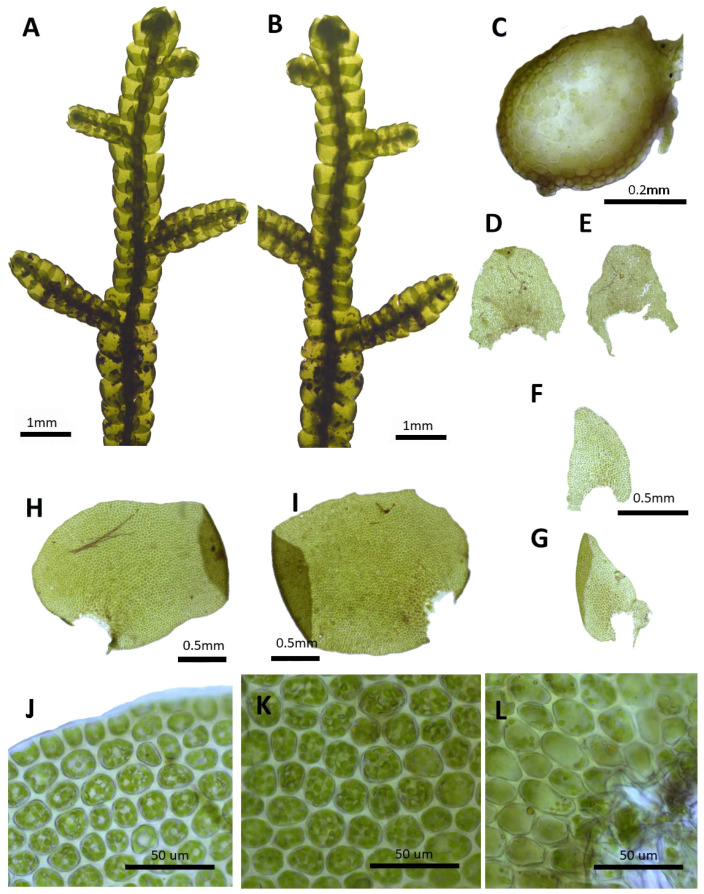
*Porella gracillima* Mitt.: (**A**) plant habit, ventral view; (**B**) plant habit, dorsal view; (**C**) cross section of the stem; (**D**,**E**) underleaves; (**F**,**G**) ventral lobes; (**H**,**I**) dorsal lobes; (**J**) marginal cells of the dorsal lobe; (**K**) middle cells of the dorsal lobe; (**L**) basal cells of the dorsal lobe. All images from Bum and S.S. Choi, 210254 (JNU).

(5) ***Porella gracillima*** Mitt., Trans. Linn. Soc. London, Bot. 3(3): 202. 1891 (Figure 6).

Description: Plants prostrate to ascending, green, yellowish brown, and brwonish green, with dimensions of 20.0–40.0 mm long and 1.5–2.0 mm wide. Stems irregularly pinnately branched; with a cross section of 0.4–0.42 × 0.32–0.35 mm; thick-walled cortex cells in 1–2 layers, with dimensions of 7.5–10.0 × 7.5–10.0 μm; and thin-walled inner cells, with dimensions of 20.0–25.0 × 20.0–25.0 μm. Rhizoids sparse or rarely few at underleaf bases. Dorsal leaf lobes imbricate, obliquely elliptical ovate, incurved to dorsal side 1/4–13 part, with dimensions of 1.12–1.37 × 1.0–1.37 mm, dorsally arcuately inserted and not decurrent, apex rounded, obtuse, and with entire. Ventral leaf lobes obliquely spreading, triangular ovate, with dimensions of 0.37–0.50 × 0.35–0.45 mm, decurrent for 0.5–1.0 of the stem width, apex obtuse, and with entre margin. Underleaves sheathing the stem near the base, shortly decurrent for 0.5–0.6 of the stem width on both sides, oliquely triangular ovate, ovate, with dimensions of 0.37–0.50 × 0.35–0.45 mm, apex rounded, obtuse, acute, reflexed with entire margin, and base area crispate. Cells in dorsal lobe middle subisodiametric to oblong, 15.0–20.0 × 15.0–20.0 μm, thin walled, trigones triangular, near apex slightly thick-walled, 10.0–12.5 × 10.0–12.5 μm, near base 25.0–30.0 × 20.0–25.0 μm, trigones concave to triangular; cuticle smooth. Oil bodies 20–25 per cells, spherical to elliptic, homogenous, and with dimensions of 2.0–3.0 × 2.0–2.5 μm. Dioicous. Gynoecia on short lateral braches arising from the main stem. Perianth with dimensions of 3.0–3.2 × 1.2–1.5 mm, eliiptical ovate, and with keels.

Habitat: This species grows on shady rocks on the slopes of mountainous areas.

Distribution: Japan, China, the Russian Far East, and India [5]. In the Korean Peninsula, it is found in Gwanmobong, Chilbosan, Myohyangsan, Ungeosusan, Ogasan, Biraebong, Sambang, Geumgangsan, Heulyeongsan, Yangamsan, Taebaeksan, Jeongseon Donggang, Deokhangsan, and Ulleungdo [6,8,11,31].

Comment: *Porella gracillima* was reported as a new species by Mitten [38] based on specimens collected in Japan. This species is very similar to *P. fauriei*, *P. vernicosa*, and *P. spinulosa* in terms of general plant size and leaf cell size but is distinguished by the absence of teeth and cilia on the dorsal and ventral lobes. *Porella spinulosa* (Steph.) S. Hatt., which is similar to this species but distinguished by the presence of teeth on the dorsal lobe and underleaves, forms one cladistic group with *P. gracillim* in the phylogenetic analysis. In the literature, Bakalin and Klimova [10] studied *Porella* in the Russian Far East and suggested that the morphological characteristics of *P. gracillim* were very similar to those of *P. vernicosa* and *P. spinulosa*. Although the cladistic analysis supported the synonymy of *P. spinulosa* with *P. gracillima*, we cannot definitely approve this synonymization because we did not see the specimen that was the basis for the GenBank sequence.

**Figure 7:**
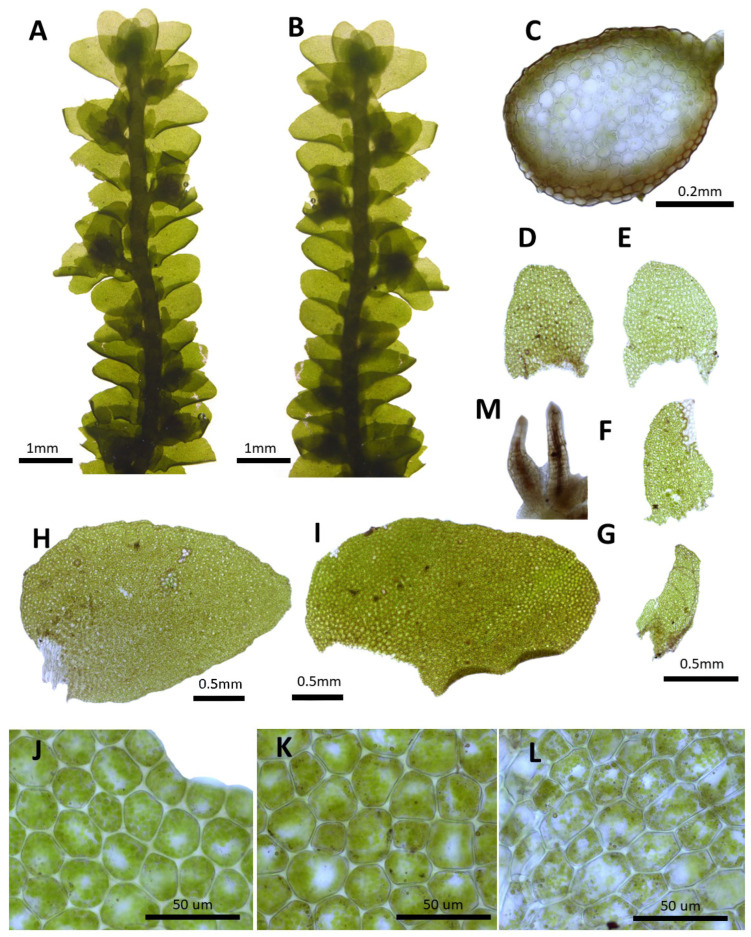
*Porella grandiloba* Lindb.: (**A**) plant habit, ventral view; (**B**) plant habit, dorsal view; (**C**) cross section of the stem; (**D**,**E**) underleaves; (**F**,**G**) ventral lobes; (**H**,**I**) dorsal lobes; (**J**) marginal cells of the dorsal lobe; (**K**) middle cells of the dorsal lobe; (**L**) basal cells of the dorsal lobe; (**M**) gynoecium. All images from Bum and S.S. Choi, 210258 (JNU).

(6) ***Porella grandiloba*** Lindb., Contr. Fl. Crypt. As. 234. 1872 (Figure 7).

Description: Plants prostrate to ascending, green, yellowish green, brownish green, and yellowish green, with dimensions of 30.0–50.0 mm long and 2.5–3.1 mm wide. Stems irregularly pinnately branched; with a cross section of 0.38–0.41 × 0.28–0.31 mm; thick-walled cortex cells in 1–2 layers, with dimensions of 10.0–12.5 × 10.0–12.5 μm; and thin-walled inner cells, with dimensions of 20.0–25.0 × 20.0–25.0 μm. Rhizoids sparse or rarely few at underleaf bases. Dorsal leaf lobes imbricate, obliquely elliptical, elliptical ovate, flatted, with dimensions of 1.4–1.6 × 1.0–1.2 mm, dorsally arcuately inserted and slightly decurrent, apex rounded, obtuse, with entire margin, and slightly crispate. Ventral leaf lobes obliquely spreading, elliptical ovate, with dimensions of 0.5–0.65 × 0.2–0.3 mm, shortly decurrent for 0.1–1.2 of the stem width, apex rounded, obtuse, rarely acute, and with entre margin. Underleaves sheathing the stem near the base, shortly decurrent for 0.1–0.2 of the stem width on both sides, oliquely elliptical, elliptical ovate, with dimensions of 0.35–0.50 × 0.31–0.35 mm, apex rounded, obtuse, and with entire margin. Cells in dorsal lobe middle subisodiametric to oblong, 30.0–37.5 × 25.0–30.0 μm, thin walled, trigones concave, near apex slightly thin-walled, 17.5–25.0 × 17.5–25.0 μm, trigones concave to triangular, near base 35.0–42.5 × 30.0–35.0 μm, trigones concave; cuticle smooth. Oil bodies 20–35 per cells, spherical to elliptic, homogenous, and with dimensions of 2.5–4.5 × 2.5–3.0 μm. (Dioicous. Androecia terminal on short lateral branches, spicate, and with 1–2 pairs of sterile leaves. Gynoecia freely produced. Perianth dorsiventrally compressed, nearly orbicular in the projection, with truncate upper part, mouth crenulate along the margin, later slightly lacerate due to capsule emerging, and with dimensions of 1.8–2.4 × 1.8–2.2 mm [10]).

Habitat: This speceis grows on the shady surfaces of rocks or on the barks of trees on mountain slopes.

Distribution: Japan, China, Taiwan, and the Russian Far East [5]. In the Korean Peninsula, it is found in Samjiyeon, Gwanmobong, Chailbong (Bujeon), Rangrimsan, Ungeosusan, Chilbosan, Sinheung, Songwon, Yangdeok, Seongcheon, Seonggan, Myohyangsan, Biraebong, Sambang, Chuaesan, Heulyeongsan, Tongcheon, Geumgangsan, Samcheon, Yangamsan, Saedaesan, Suyangsan, Seoraksan, Odaesan, Taebaeksan, Sobaeksan, Songnisan, Deogyusan, Jirisan, Ulleungdo, and Jejudo [6,8,11,31].

Comment: *P. grandiloba* was reported as a new species by Lindberg [39] based on specimens collected in North Sakhalin (Due Bay) by Glehn. It is a relatively common taxon in Korea and is characterized by the dorsal and ventral lobes, with underleaves being the entire margin, which is shortly decurrent for the stem. *P. pinnata* L. has been reported to be distributed in Ogasan, Songwon, and Chuaesan in North Korea and is very similar to *P. grandiloba* in dorsal and ventral lobe shape; however, considering its global distribution [5], it was excluded because it is highly likely to be a misidentification of *P. grandiloba* [11].

**Figure 8:**
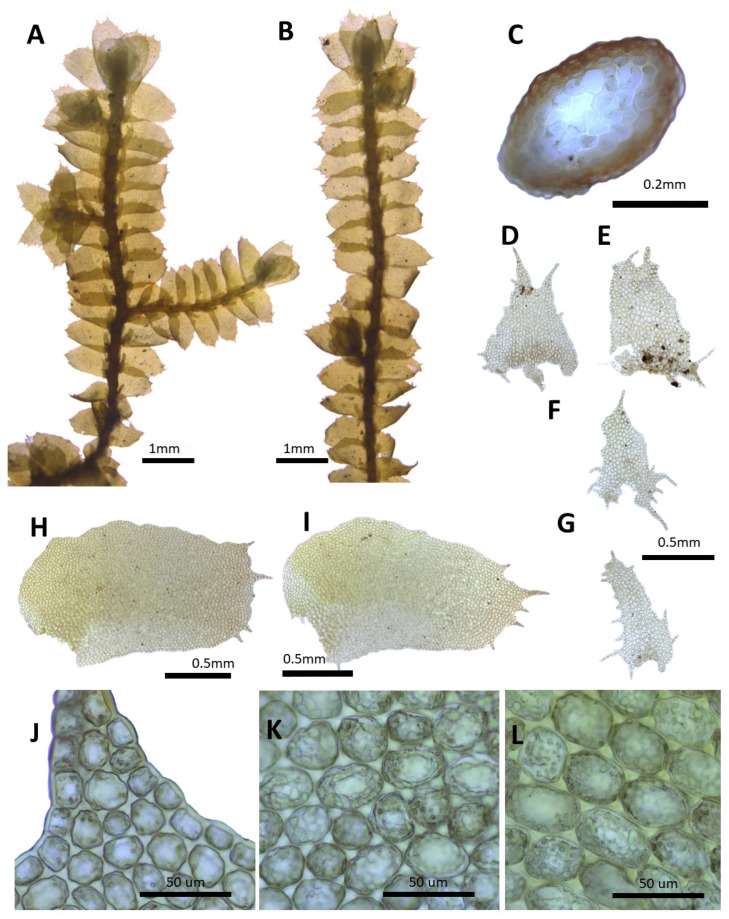
*Porella japonica* (Sande Lac.) Mitt.: (**A**) plant habit, ventral view; (**B**) plant habit, dorsal view; (**C**) cross section of the stem; (**D**,**E**) underleaves; (**F**,**G**) ventral lobes; (**H**,**I**) dorsal lobes; (**J**) marginal cells of the dorsal lobe; (**K**) middle cells of the dorsal lobe; (**L**) basal cells of the dorsal lobe (Jeju). All images from Bum and S.S. Choi, 120104 (JNU).

**Figure 9:**
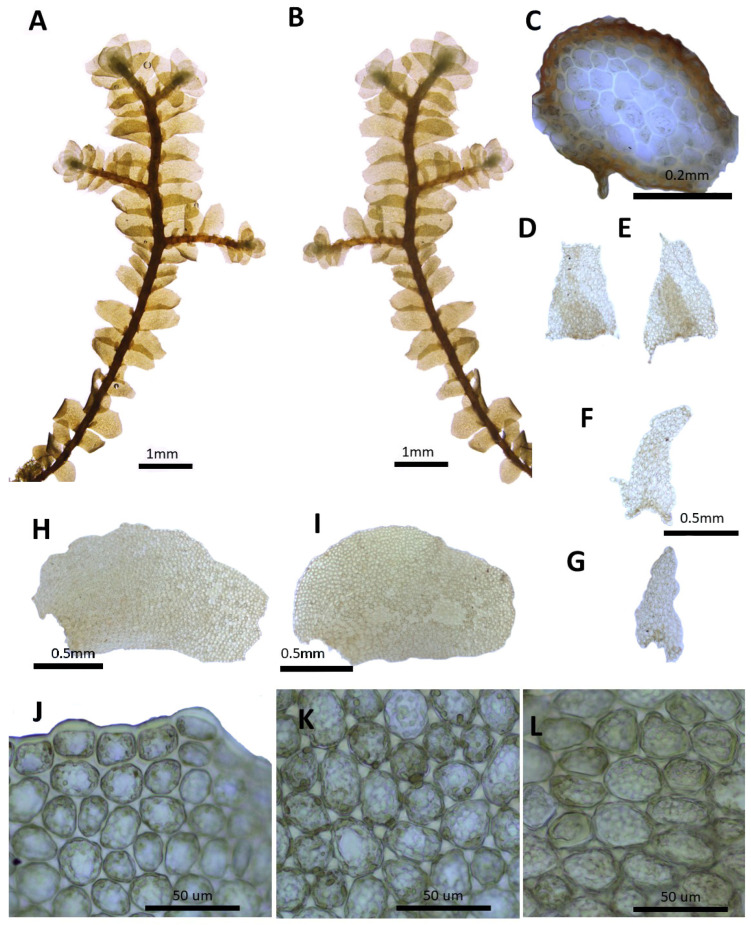
*Porella japonica* (Sande Lac.) Mitt.: (**A**) plant habit, ventral view; (**B**) plant habit, dorsal view; (**C**) cross section of the stem; (**D**,**E**) underleaves; (**F**,**G**) ventral lobes; (**H**,**I**) dorsal lobes; (**J**) marginal cells of the dorsal lobe; (**K**) middle cells of the dorsal lobe; (**L**) basal cells of the dorsal lobe (Jeju). All images from Bum and S.S. Choi, 201080a (JNU).

(7) ***Porella japonica*** (Sande Lac.) Mitt., Trans. Linn. Soc. London, Bot. 3(3): 202. 1891 (Figure 8 and Figure 9).

Basionym: *Madotheca japonica* Sande Lac., Syn. hepat. jav.: 105, 1856 [1857].

Description: Plants ascending, brownish green, light brown, light green, and yellowish green, with dimensions of 20.0–40.0 mm long and 1.8–2.9 mm wide. Stems irregularly pinnately branched; with a cross section of 0.24–0.26 × 0.15–0.17 mm; thick-walled cortex cells in 1–2 layers, with dimensions of 7.5–12.5 × 7.5–12.5 μm; and thin-walled inner cells with dimensions of 17.5–25.0 × 17.5–25.0 μm. Rhizoids sparse or rarely few at underleaf bases. Dorsal leaf lobes imbricate, obliquely elliptical, flatted, with dimensions of 1.2–1.4 × 0.6–0.8 mm, dorsally arcuately inserted and slightly decurrent, apex rounded, obtuse, with 2–6 teeth, ciliate or with entire margin, and slightly crispate. Ventral leaf lobes obliquely spreading, elliptical ovate, narrowly elliptical, with dimensions of 0.35–0.65 × 0.20–0.25 mm, shortly decurrent for the stem, apex acute, obtuse, with 5–12 teeth or ciliate, and entire margin. Underleaves sheathing the stem near the base, shortly decurrent for the stem on both sides, oliquely ovate, triangular ovate, with dimensions of 0.5–0.75 × 0.25–0.35 mm, apex obtuse, acute, and with 1–4 tooth. Cells in dorsal lobe middle subisodiametric to oblong, 25.0–30.0 × 20.0–25.0 μm, thin walled, trigones convex, near apex slightly thin-walled, 17.5–25.0 × 17.5–25.0 μm, trigones convex, near base 30.0–37.5 × 22.5–27.5 μm, trigones convex; cuticle smooth. Oil bodies 10–25 per cells, spherical to elliptic, homogenous, and with dimensions of 2.5–4.0 × 2.0–3.0 μm.

Habitat: This species grows on the shady surfaces of rocks or at the bases of trees in mountainous areas or valleys

Distribution: Japan, China, Taiwan, Vietnam, India, Malaysia, and the Philippines [5]. In the Korean Peninsula, it is found in Hallasan and in Gotjawal on Jeju Island [6,8,11,31].

Comment: *Porella japonica* was first reported by Sande Lacoste [40], based on specimens collected in Japan. It was later transferred to the genus *Porella* by Mitten [38]. *P. japonica* is characterized by the presence or absence of teeth on the margins of the ventral and dorsal lobes and triangular underleaves with truncated and commonly bitoothed apices. Individual plants without teeth on their margins are sometimes misidentified as *P. grandiloba* or *P. gracillima*.

Specimens identified as *P. japonica* in areas other than Jeju Island did not form a single cladistic group with the Jeju Island specimens. As a result of morphological reexamination of these specimens, they were distinguished by the following characteristics: concave apices, narrow plant width, and relatively small cell size. Accordingly, the peninsular individuals were described as a new taxonomic group, *P. chulii* sp. nov.

**Figure 10:**
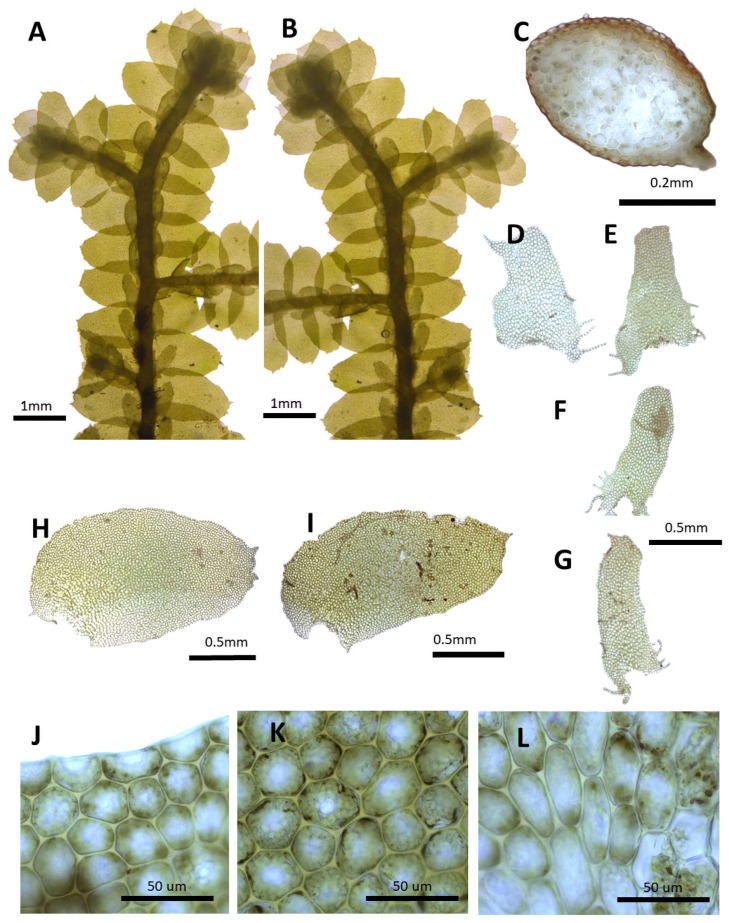
*Porella oblongifolia* S. Hatt.: (**A**) plant habit, ventral view; (**B**) plant habit, dorsal view; (**C**) cross section of the stem; (**D**,**E**) underleaves; (**F**,**G**) ventral lobes; (**H**,**I**) dorsal lobes; (**J**) marginal cells of the dorsal lobe; (**K**) middle cells of the dorsal lobe; (**L**) basal cells of the dorsal lobe. All images from Bum and S.S. Choi, 170870 (JNU).

(8) ***Porella oblongifolia*** S. Hatt., J. Jap. Bot. 19: 200. f. 20. 1943 (Figure 10).

Description: Plants prostrate to ascending, yellowish green, brownish green, green, and yellowish brownn, with dimensions of 30.0–40.0 mm long and 3.0–3.5 mm wide. Stems irregularly pinnately branched; with a cross section of 0.40–0.45 × 0.25–0.30 mm; thick-walled cortex cells in 2–3 layers, with dimensions of 10.0–12.5 × 10.0–12.5 μm; and thin-walled inner cells with dimensions of 25.0–30.0 × 25.0–30.0 μm. Rhizoids sparse or rarely few at underleaf bases. Dorsal leaf lobes imbricate, obliquely elliptical, oblong, flatted, with dimensions of 1.7–2.3 × 1.1–1.3 mm, dorsally arcuately inserted and slightly decurrent, apex rounded, obtuse, with 1–3 teeth or ciliate near the apex, and entire margin near the base. Ventral leaf lobes obliquely spreading, narrowly elliptical, with dimensions of 0.6–0.9 × 0.3–0.5 mm, shortly decurrent for the stem, apex obtuse, rounded, with entire margin near the apex, and with 3–4 teeth or ciliate near the base. Underleaves sheathing the stem near the base, decurrent for 0.8–1.0 of the stem width, oliquely ovate, triangular ovate, with dimensions of 0.6–0.9 × 0.3–0.5 mm, apex obtuse, and with a margin of 3–4 teeth. Cells in the middle dorsal lobe subisodiametric to oblong, with dimensions of 30.0–37.5 × 25–32.5 μm, thin walled, and trigones concave; slightly thin-walled near the apex, with dimensions of 22.5–27.5 × 22.5–27.5 μm and trigones concave; and with dimensions of 37.5–50.0 × 22.5–27.5 μm near the base, with triangular trigones; cuticle smooth. Oil bodies 15–30 per cells, spherical to elliptic, homogenous, and with dimensions of 2.5–5.0 × 2.0–3.0 μm.

Habitat: This species grows on the shady surfaces of rocks in mountains or at the bases of trees.

Distribution: Japan, China, and the Russian Far East [5]. In the Korean Peninsula, it is found in Chilbosan, Seonggan, Taebaeksan, Deogyusan, Wolaksan, Eungboksan, Manghyangsan, and Taebaeksan [6,8,11,31].

Comment: *Porella oblongifolia* was described as a new species by Hattori [41] based on specimens collected from Mt. Mitake near Tokyo, Japan. It is distinguished from *P. grandiloba* and *P. vernicosa* by the fact that the dorsal lobe is flat rather than curved inward, although the apex is toothed. The shape of the dorsal lobe is similar to that of *P. japonica*; however, the size of the dorsal lobe is approximately twice as large, and the size of the middle cell of the dorsal lobe is 30.0–37.5 μm. *P. tosana*, similar to *P. oblongifolia*, was first recorded on the Korean Peninsula by Stephani [26]. However, considering that the distribution area of *P. tosana* is a taxon distributed in tropical and subtropical regions, it was judged to be highly likely to be a misidentification of the similar *P. oblongifolia* and was therefore excluded.

**Figure 11:**
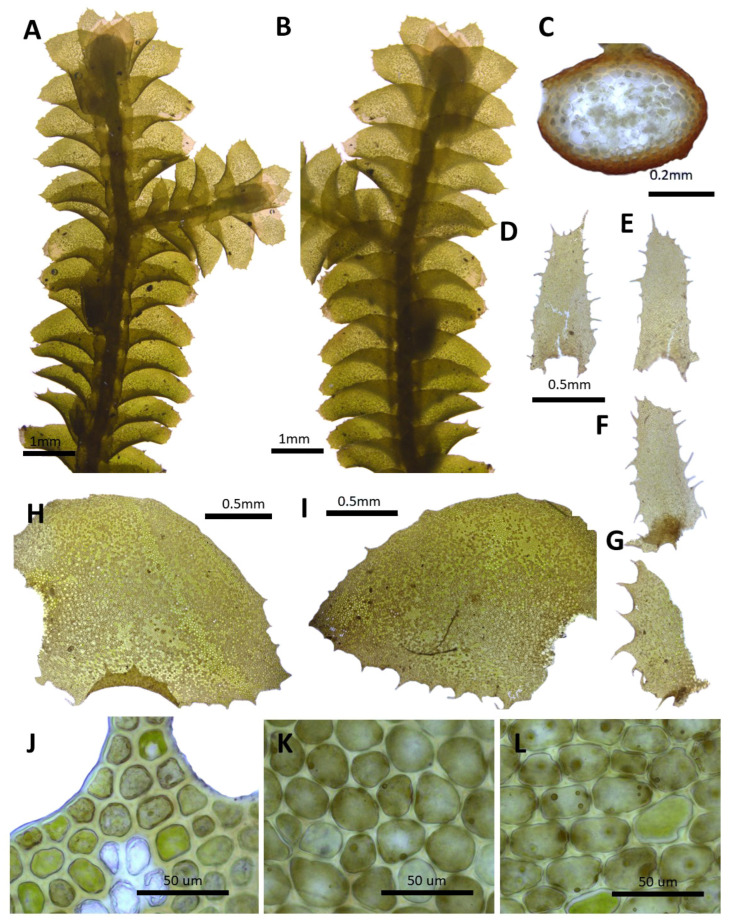
*Porella stephaniana* (C. Massal.) S. Hatt.: (**A**) plant habit, ventral view; (**B**) plant habit, dorsal view; (**C**) cross section of the stem; (**D**,**E**) underleaves; (**F**,**G**) ventral lobes; (**H**,**I**) dorsal lobes; (**J**) marginal cells of the dorsal lobe; (**K**) middle cells of the dorsal lobe; (**L**) basal cells of the dorsal lobe. All images from Bum and S.S. Choi, 170093 (JNU).

(9) ***Porella stephaniana*** (C. Massal.) S. Hatt., J. Hattori Bot. Lab. 5: 81. 1951 (Figure 11).

Basionym: *Madotheca stephaniana* C.Massal., Hepat. Shen-si: 23, 1897.

Description: Plants prostrate to ascending, brownish green, dark green, or green, with dimensions of 50.0–100.0 mm long and 3.1–3.8 mm wide. Stems irregularly pinnately branched; with a cross section of 0.38–0.41 × 0.25–0.35 mm; thick-walled cortex cells in 2–3 layers, with dimensions of 7.5–10.0 × 5.0–7.5 μm; and thin-walled inner cells with dimensions of 20.0–25.0 × 20.0–25.0 μm. Rhizoids sparse or rarely few at underleaf bases. Dorsal leaf lobes imbricate, obliquely triangular ovate, upper margin of the side curved, lower margin of the side straight, with dimensions of 1.6–2.3 × 1.2–1.7 mm, dorsally arcuately inserted and slightly decurrent, apex acute, with a margin of 10–25 teeth, and slightly crispate near the base. Ventral leaf lobes obliquely spreading, narrowly elliptical, narrowly oblong, with dimensions of 0.7–1.0 × 0.2–0.4 mm, shortly decurrent for the stem, apex obtuse, rounded, and with a margin of 6–10 teeth. Underleaves sheathing the stem near the base, decurrent for 1.0–1.1 of the stem width, oliquely narrowlly oblong, narrowly elliptical, with dimensions of 0.7–1.0 × 0.2–0.4 mm, apex obtuse, rounded, and with a margin of 10–20 teeth. Cells in the middle dorsal lobe subisodiametric to oblong, with dimensions of 25.0–37.5 × 25.0–30.0 μm, thin walled, and trigones convex; thick-walled near the apex, with dimensions of 12.5–17.5 × 12.5–17.5 μm and trigones convex; and with dimensions of 32.2–42.5 × 25.0–32.5 μm near the base, with convex trigones; cuticle smooth. Oil bodies 20–30 per cells, spherical to elliptic, homogenous, and with dimensions of 2.0–5.0 × 2.0–3.0 μm.

Habitat: This species grows on the shady and moist surfaces of rocks in limestone areas.

Distribution: Japan and China [5]. On the Korean Peninsula, it is found on Donggang River (Jeongseon-gun) and Wangpicheon Stream (Uljin-gun) [11].

Comment: *Porella stephaniana* was described as a new species, *Madotheca stephaniana*, by Massalongo [42] based on specimens collected in Shaanxi Province, China. It was later transferred to the genus *Porella* by Hattori [16]. Choi et al. [36] reported this species as a new record from the Korean Peninsula based on specimens collected in the Donggang Limestone area of Jeongseon-gun. It is distinguished from other taxa in the genus by the oblique triangular-ovate shape of the dorsal lobes and the short teeth on the dorsal lobe margins.

**Figure 12:**
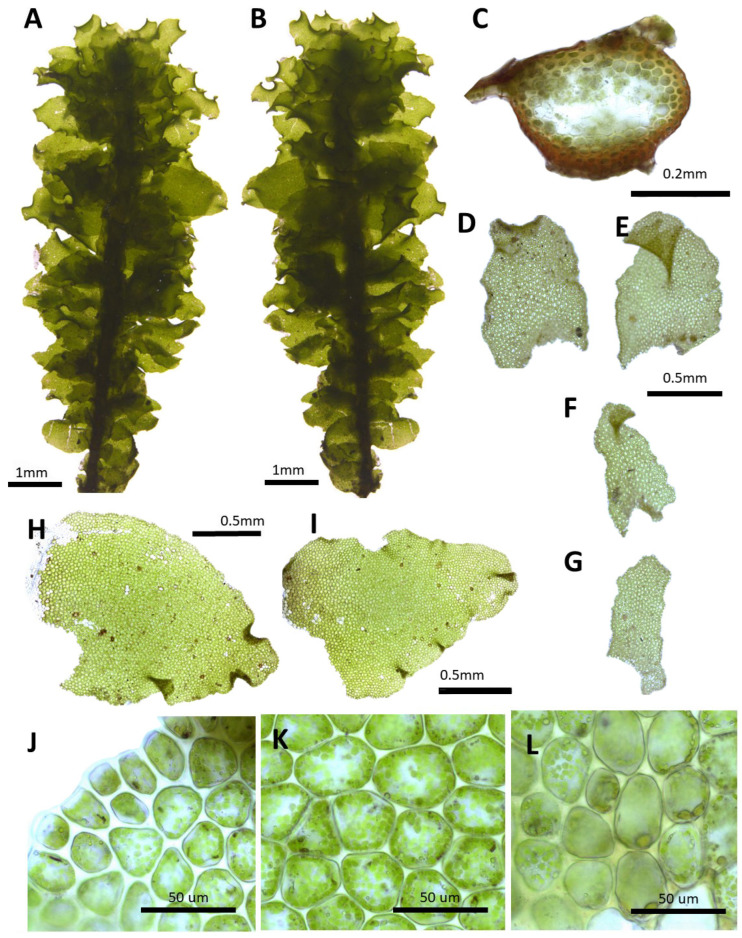
*Porella ulophylla* (Steph.) S. Hatt.: (**A**) plant habit, ventral view; (**B**) plant habit, dorsal view; (**C**) cross section of the stem; (**D**,**E**) underleaves; (**F**,**G**) ventral lobes; (**H**,**I**) dorsal lobes; (**J**) marginal cells of the dorsal lobe; (**K**) middle cells of the dorsal lobe; (**L**) basal cells of the dorsal lobe. All images from Bum and S.S. Choi, 210014 (JNU).

(10) ***Porella ulophylla*** (Steph.) S. Hatt., Bull. Tokyo Sci. Mus. 11: 92. 1944 (Figure 12).

Basionym: *Madotheca ulophylla* Steph., Bull. Herb. Boissier 5: 97. 1897.

Description: Plants prostrate to ascending, green, brownish green, or brownish yellow, with dimensions of 20.0–40.0 mm long and 3.2–3.8 mm wide. Stems irregularly pinnately branched; with a cross section of 0.33–0.36 × 0.25–0.30 mm; thick-walled cortex cells in 2–3 layers, with dimensions of 7.5–10.0 × 7.5–10.0 μm; and thin-walled inner cells with dimensions of 20.0–25.0 × 20.0–25.0 μm. Rhizoids sparse or rarely few at underleaf bases. Dorsal leaf lobes imbricate, obliquely triangular ovate, arcuately inserted, with dimensions of 1.6–2.0 × 0.8–1.2 mm, dorsally arcuately inserted and not decurrent, apex rounded, obtuse, strongly crispate with entire margin, and wrinkles in the margin. Ventral leaf lobes obliquely spreading, narowlly elliptic to lingulate, with dimensions of 0.4–0.7 × 0.2–0.3 mm, not decurrent, apex rounded, obtuse, and crispate to wrinkeles with entire margin. Underleaves sheathing the stem near the base, decurrent for 1.0–1.5 of the stem width on both sides, ovate, triangular ovate, with dimensions of 0.5–0.6 × 0.3–0.4 mm, apex, obtuse, crispate, amd with wrinkles with entire margin. Cells in the middle dorsal lobe subisodiametric to oblong, with dimensions of 32.5–40.0 × 25.0–30.0 μm, thin walled, and trigones triangular; slightly thick-walled near the apex, with dimensions of 15.0–20.0 × 15.0–20.0 μm; and with dimensions of 35.0–45.0 × 30.0–37.5 μm near the base, with triangular to convex trigones; cuticle smooth. Oil bodies 20–30 per cells, spherical to elliptic, homogenous, and with dimensions of 2.5–5.0 × 2.5–3.0 μm. Dioicous. Gynoecia on short lateral braches, perianth with dimensions of 2.0–2.4 × 1.2–1.5 mm, eliiptical obovate, with 3–4 keels, and mouth with teeth.

Habitat: This species grows on tree barks and rarely on the surfaces of rocks.

Distribution: Japan, China, and the Russian Far East [5]. On the Korean Peninsula, it is found on Leemyeongsu, Songjinsan, Myohyangsan, Seonggan, Biraebong, Tongcheon, Chuaesan, Heulyeongsan, Geumgangsan, Saedaesan, Suyangsan, Gaeseong, Jirisan, Jeju Island, Jirisan, and Deogyusan [6,8,11,31].

Comment: In 1897, Stephani described a new species as *Madotheca ulophylla* based on specimen 15034, which was collected by U. Faurie from Mt. Chichubu, Japan [24]. Later, Hattori [15] transferred it to the genus *Porella*. This species was classified into another genus, *Macvicaria*, because of its highly crisp dorsal lobes [16]; however, it is currently classified within the genus *Porella* [2,3].

**Figure 13:**
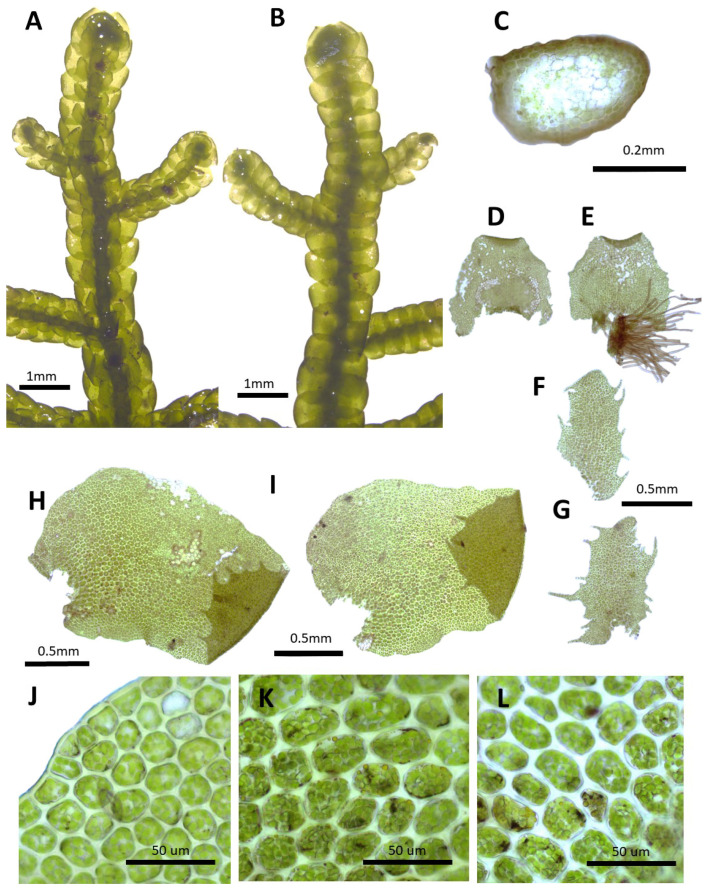
*Porella vernicosa* Lindb.: (**A**) plant habit, ventral view; (**B**) plant habit, dorsal view; (**C**) cross section of the stem; (**D**,**E**) underleaves; (**F**,**G**) ventral lobes; (**H**,**I**) dorsal lobes; (**J**) marginal cells of the dorsal lobe; (**K**) middle cells of the dorsal lobe; (**L**) basal cells of the dorsal lobe. All images from Bum and S.S. Choi, 210234 (JNU).

(11) ***Porella vernicosa*** Lindb., Contr. Fl. Crypt. As. 223. 1872[1873] (Figure 13).

Description: Plants prostrate to ascending, light green, green, yellowish green, or brownish green, with dimensions of 20.0–30.0 mm long and 1.5–1.9 mm wide. Stems irregularly pinnately branched; with a cross section of 0.35–0.4 × 0.30–0.35 mm; thick-walled cortex cells in 2–3 layers, with dimensions of 10.0–12.0 × 10.0–12.0 μm; and thin-walled inner cells with dimensions of 20.0–25.0 × 20.0–25.0 μm. Rhizoids sparse or rarely few at underleaf bases. Dorsal leaf lobes imbricate, obliquely elliptical ovate, strongly incurved to dorsal side 1/3–1/2 part, arcuately inserted, with dimensions of 0.8–1.2 × 0.5–0.8 mm, dorsally arcuately inserted and not decurrent, apex rounded, obtuse, slightly crispate to entire, with 5–15 teeth, and ventral margin sparsely toothed to entire. Ventral leaf lobes obliquely spreading, lingulate to narrowly oblong, with dimensions of 0.5–0.6 × 0.2–0.4 mm, not decurrent, apex acute, obtuse, with 10–20 teeth, and densely to sparsely toothed. Underleaves sheathing the stem near the base, decurrent for 1.0–1.2 of the stem width on both sides, triangular ovate, deflexed, with dimensions of 0.5–0.65 × 0.3–0.5 mm, an obtuse apex, and acute with entire margin for 1–4 teeth. Cells in the middle dorsal lobe subisodiametric to oblong, with dimensions of 22.5–32.5 × 17.5–25.0 μm, thin walled, and convex to triangular trigones; slightly thick-walled near the apex, triangular trigones, and with dimensions of 12.5–17.5 × 12.5–17.5 μm; and dimensions of 25.0–35.0 × 20.0–25.0 μm near the base, with convex trigones; cuticle smooth. Oil bodies 15–30 per cells, spherical to elliptic, homogenous, and with dimensions of 2.5–3.5 × 2.0–3.0 μm.

Habitat: This species grows on the shady surfaces of rocks on mountain slopes or valleys or at the bases of trees.

Distribution: Japan, China, and the Russian Far East. On the Korean Peninsula, it is found on Rangrimsan, Ungeosusan, Chilbosan, Myohyangsan, Seonggan, Ogasan, Biraebong, Sinyang, Tongcheon, Chuaesan, Heulyeongsan, Geumgangsan, Saedaesan, Suyangsan, Seoraksan, Odaesan, Taebaeksan, Sobaeksan, Eungboksan, Wolaksan, Sokrisan, Ulleungdo, Deogyusan, Jirisan, Oenarodo, and Jejudo [6,8,11,31].

Comment: *Porella vernicos* was described as a new species by Lindberg [39] based on specimens collected in Nagasaki, Japan. It is distinguished from other species within the genus by the characteristic features of the dorsal lobe having many teeth on the leaf margin and the tip being severely curved inward.

**Figure 14:**
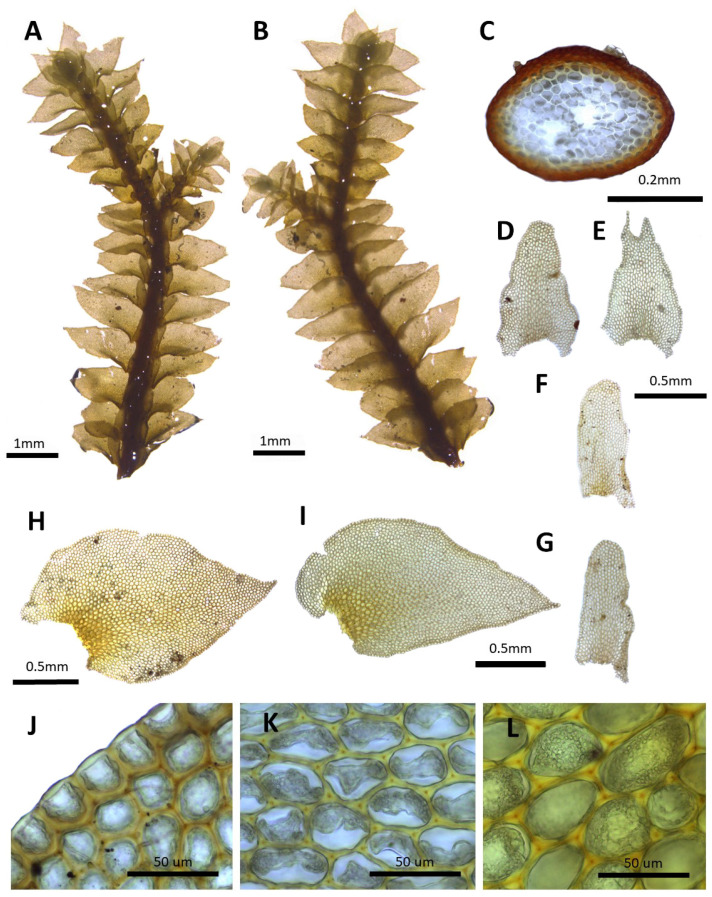
*Porella koreana* H.M. Bum, sp. nov.: (**A**) plant habit, ventral view; (**B**) plant habit, dorsal view; (**C**) cross section of the stem, (**D**,**E**) underleaves; (**F**,**G**) ventral lobes; (**H**,**I**) dorsal lobes; (**J**) marginal cells of the dorsal lobe; (**K**) middle cells of the dorsal lobe; (**L**) basal cells of the dorsal lobe. All images from S.J. Park, 13899 (HIBR).

(12) ***Porella koreana*** H.M. Bum, S.J. Park, Bakalin and S.S. Choi, sp. nov. (Figure 14).

Holotype: Gyeongsangbuk-do, Cheongsong-gun, wind hall, 13 Oct 2015, S.J. Park, 13899 (HIBR).

Description: Plants prostrate to ascending, brownish green, light brown, and green, with dimensions of 30.0–50.0 × 2.5–3.5 mm. Stems irregularly pinnately branched; with dimensions of 0.5–0.6 × 0.30–0.35 μm wide; thick-walled cortex cells in 2–3 layers, with dimensions of 12.5–25.0 × 7.5–10.0 μm; and thin-walled inner cells with dimensions of 25.0–37.5 × 15.0–30.0 μm. Rhizoids sparse or rarely few at underleaf bases. Dorsal leaf lobes imbricate, contiguous, obliquely triangular ovate, with dimensions of 1.5–1.9 × 0.8–1.2 mm, dorsally arcuately inserted and barely decurrent, entire margin, and an acute apex. Ventral leaf lobes obliquely spreading, narrow oblong, oblong ovate, with dimensions of 0.7–1.0 × 0.25–0.37 mm, decurrent for 0.3–0.5 of the stem width, rounded apex, and obtuse with crispate margin. Underleaves mostly squarrose, sheathing the stem near the base, narrowly ovate, decurrent for 1.0–1.2 of the stem width on both sides, obtuse, bilobed near the apex, with dimensions of 0.7–1.0 × 0.3–0.62 mm, and mostly entire to crispate. Cells in the middle dorsal lobe with dimensions of 30.0–37.5 × 25–30 μm, subisodiametric to shortly oblong, thin walled, and triangular to convex trigones; thick-walled near the apex, triangular trigones, and with dimensions of 25.0–30.0 × 25.0–30.0 μm; and with dimensions of 37.5–50.0 × 30.0–37.5 μm near the base, with convex trigones; cuticle smooth. Oil bodies 15–30 per cells, spherical to elliptic, homogenous, and with dimensions of 2.5–3.5 × 2.0–3.0 μm.

Korean name: Seonsejulikki.

Habitat: This species is found on the shady surfaces of rocks in wind holes.

Distribution: Korea (Cheongsong-gun) and Japan (Shikoku).

Comment: The specimen collected from Cheongsong-gun (specimen number Park 13899) was initially identified as *P. caespitans*. However, the width of the plant is 2.5–3.5 mm, and the apex of the dorsal lobe is acute, distinguishing it from *P. caespitans*, which is 2.5–3.8 mm in width and has an acuminate apex of the dorsal lobe. Considering these features, a phylogenetic analysis was performed, the results of which revealed that it does not form a single clade with *P. caespitans* individuals. It was judged to be a new species, as it can be distinguished from similar taxa by the acute apex of the dorsal leaf lobe and the triangular or convex trigones. In addition, this specimen is similar to *P. stephaniana* in that the apices of the dorsal lobe are acute; however, it can be distinguished by the entire margin on the dorsal lobe.

Hara [37] described and illustrated *P. subobtusa* in the *Porella* of Shikoku, Japan, and it appears that it was recorded through misidentification without the recognition of the new taxon. In particular, after Hattori [19] observed the type specimen of *P. subobtusa*, he noted that the illustration expressed by Hara [37] was incorrectly described because the type specimen was not observed. The original description and illustration of the type specimen of *P. subobtusa* revealed that the apex of the dorsal lobe is not acute but obtuse. The latter may indicate the occurrence of a new species in Japan.

The Korean name is Seonsejulikki in honor of Professor Byeong-Yun Sun, who contributed actively to supporting the research on bryophytes.

**Figure 15:**
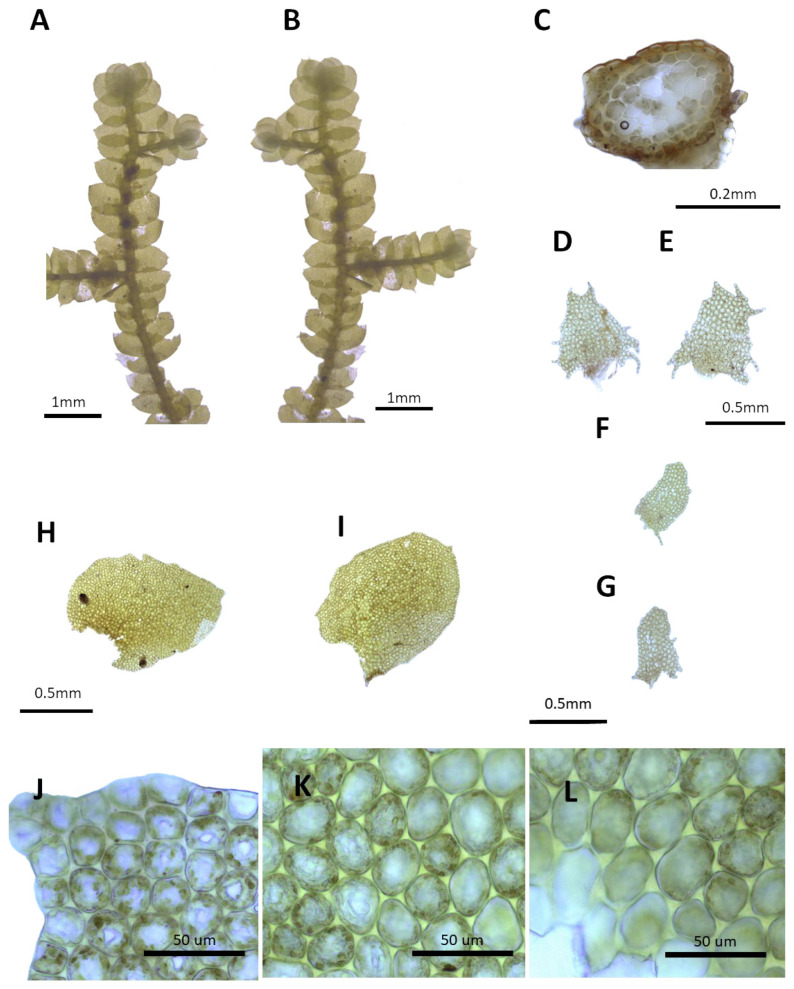
*Porella chulii* H.M. Bum, sp. nov.: (**A**) plant habit, ventral view; (**B**) plant habit, dorsal view; (**C**) cross section of the stem; (**D**,**E**) underleaves; (**F**,**G**) ventral lobes; (**H**,**I**) dorsal lobes; (**J**) marginal cells of the dorsal lobe; (**K**) middle cells of the dorsal lobe; (**L**) basal cells of the dorsal lobe. All images from Bum and S.S. Choi, 170090 (HIBR).

(13) ***Porella chulii*** H.M. Bum, S.J. Park, Bakalin and S.S. Choi, sp. nov (Figure 15).

Holotype: Gangwon-do, Taebaek-si, Mt. Taebaeksan, Gyeomryoungso, 15 Apr 2017, H.M. Bum and S.S. Choi, 170090 (HIBR).

Description: Plants prostrate to ascending; rarely erect in dense patches; yellowish green, green, brown, and yellowish brown, and with dimensions of 10.0–30.0 × 1.37–2.25 mm. Stems irregularly pinnately or bipinnately branched, with dimensions of 0.4–0.45 × 0.30–0.35 μm wide. Rhizoids virtually absent. Dorsal leaf lobes imbricate, contiguous, ovate, slightly incurved at the apex, with dimensions of 0.8–1.2 × 0.6–1.0 mm, dorsally arcuately inserted and barely decurrent, entire margin, and an acute apex. Ventral leaf lobes slightly obliquely spreading, narrow oblong, oblong ovate, with dimensions of 0.25–0.37 × 0.20–0.25 mm, and decurrent for 0.1–0.2 of the stem width. Underleaves sheathing the stem near the base, decurrent for 0.1–0.2 of the stem width on both sides, obtuse, acute near the apex, with dimensions of 0.3–0.5 × 0.25–0.35 mm, and mostly ciliate. Cells in the middle dorsal lobe with dimensions of 20.0–25.0 × 20.0–25.0 μm, subisodiametric to shortly oblong, triangular to slightly convex trigones; thin-walled near the apex, triangular or concave trigones, and with dimensions of 12.5–17.5 × 12.5–17.5 μm; and with dimensions of 30.0–37.5 × 22.5–27.5 μm near the base, with triangular to convex trigones; cuticle smooth. Androecia. Gynoecia not seen.

Korean name: Chulikki

Habitat: This species grows on the shady surfaces of rocks in valleys.

Distribution: Taebaeksan (Geomryongso), Cheongsong, Deogyusan, and Wolaksan.

Comment: *P. chulii* was initially identified as *P. japonica* because its plant size and the apices of its dorsal lobes and underleaves are similar to those of *P. japonica*. However, *P. chulii* has a relatively narrow plant width of 1.37–2.25 mm, slightly concave underleaves, and a middle cell size of the dorsal lobes of 25.0–37.5 × 25.0–37.5 μm. *P. japonica*, which is similar to this species, has a relatively wide plant width of 1.80–2.90 mm, flat underleaves, and a middle cell size of 25.0–30.0 × 20.0–25.0 μm, which differentiates it from *P. chulii*. In addition, the Bayesian phylogenetic tree revealed that *P. chulii* does not form a single clade with *P. japonica;* however, it is a different clade, supporting the idea that it is a new taxon.

The Korean name is Chulikki in honor of the late Dr. Chul-Hwan Kim, who contributed to research on bryophytes in Korea.

### Excluded or Doubtful Species from the List in the Korean Peninsula

(1) ***Porella acutifolia*** ssp. **tosana** (Steph.) S. Hatt., J. Hattori Bot. Lab. 44: 100, 1978.

Basionym: *Madotheca tosana* Steph., Bull. Herb. Boissier 5 (2): 97, 1897.

This species was first recorded in Korea by Stephani [26]; however, there have been no records of its discovery since then. This species is quite similar to *P. oblongifolia* in that the dorsal lobe leaf margins are serrated; therefore, it was excluded due to the high possibility of misidentification.

(2) ***Porella platyphylla*** (Mitt.) Pfeiff., Fl. Niederhessen 2: 234, 1855 (Pfeiffer 1855).

Basionym: *Jungermannia platyphylla* L., Sp. Pl. 1: 1134, 1753 [12].

This species is known to be distributed in Myohyangsan, Chuaesan, and Yangdeok in North Korea [8]; however, the shapes of the dorsal lobe and ventral lobe are very similar to those of *P. densifolia*. We did not observe the specimen and suspect that it was absent. However, the distribution of *P. densifolia* in North Korea is less likely than that of *P. platyphylla*. In addition, *P. platyphylla* is locally abundant in some areas of the southern Far East of Russia; thus, its distribution may be expected in North Korea. At the current stage, we cannot definitively confirm or reject this taxon from the Korean flora.

(3) ***Porella perrottetiana*** (Mont.) Trevis., Mem. Reale Ist. Lombardo Sci. (Ser. 3), C. Sci. Mat. 4 (13): 408, 1877.

Basionym: *Madotheca perrottetiana* Mont., Ann. Sci. Nat. Bot. (sér. 2) 18: 15, 1842.

The first distribution record for this species was recorded by Hong [43]; it was later recorded as being distributed in Korea [5,44], but we have not confirmed it. The plant is large, measuring at 4.0–4.5 mm in width and 100 mm in length, and the characteristic of having serrated margins on the dorsal lobes is similar to that of *P. oblongifolia*; therefore, it is very likely to be a misidentification. The worldwide distribution includes Japan (Honshu, Shikoku, Kyushu, Yakushima Island), Taiwan, India, Myanmar, Vietnam, Sri Lanka, and the Philippines [5].

(4) ***Porella pinnata*** L., L., Sp. Pl. 1: 1106, 1753.

This species is known to be distributed in Ogasan, Songwon, and Chuaesan in North Korea [8]; however, considering that its worldwide distribution area is limited to Europe and North America [5], it was excluded because it is highly likely to be a misidentification of *P. grandiloba* [11].

(5) ***Porella spinulosa*** (Steph.) S. Hatt., J. Hattori Bot. Lab. 33: 74, 1970.

Basionym: *Madotheca spinulosa* Steph., Sp. Hepat. (Stephani) 6: 529, 1924.

The status of this species is doubtful (cf. [10]). We did not see the specimen on which this report was based for Korea. Moreover, the distribution of *P. vernicosa* in Korea is less likely than that of *P. spinulosa*. Additional morphological and molecular phylogenetic studies are needed for this species in the future. The GenBank accessions of *P. spinulosa* included in our phylogenetic tree show that it should be treated as the late synonym of *P. gracillima*, but this looks as doubtful from a morphological point of view when comparing the types of species (cf. [10]).

(6) ***Porella subobtusa*** (Steph.) S. Hatt., J. Jap. Bot. 20: 111. 1944.

Basionym: *Madotheca subobtusa* Steph., Sp. Hepat. (Stephani) 4: 311, 1910

The first distribution record for this species was provided by Hong [43], and it was later recorded as being distributed in Korea [5,44]; however, we have not confirmed its presence.

This species was first described as *Madotheca subobtusa* by Stephani [26] using specimen 646, which was collected by Father U. Faurie in Tsurugizan, Japan, in 1900. Later, Hattori [15] transferred it to *Porella subobtusa* (Steph.) S. Hatt. and changed it to *Porella setigera* var. *subobtusa* (Steph.) S. Hatt. [45]. However, Hara [37] did not agree with the view of Hattori [45], re-evaluated *P. subobtusa* as a species, and described and illustrated it as a taxon that is acute at the apex of the dorsal lobes. Hara’s *P. subobtusa* is likely misidentified as *P. koreana*. Furthermore, Hattori [19] observed the holotype of U. Faurie and described and illustrated that the tip of the dorsal lobe is obtuse, and Hattori [19] suggested that Hara [37] may have been confused because he did not observe the holotype. Combining the observation drawings and research records of the holotype specimen, it can be seen that *P. subobtusa* has an obtuse apex of the dorsal lobes, as described by Stephani [26]. Therefore, *P. subobtusa* has obtuse or subobtuse dorsal lobes, but not the characteristic of an acute apex of the dorsal lobes.

## 4. Discussion

The genus *Porella* is widely distributed throughout the world in the temperate to tropical areas of the Northern Hemisphere, with the largest number of taxa distributed in East Asia. The Korean Peninsula is the center of temperate Pacific Asia, and various taxa are distributed in China, Japan, and the Russian Far East. The genus *Porella* has many taxa with geographically limited distribution areas, and research on this genus is difficult be cause of the limited gametophyte identification characteristics. Therefore, a taxonomic study on the genus *Porella* on the Korean Peninsula was conducted.

Among the 17 taxa recorded in the literature, 6 taxa (*P. tosana*, *P. platyphylla*, *P. perrottetiana*, *P. pinnata*, *P. subobtusa*, and *P. spinulosa*) with a low probability of distribution in Korea or no probability of distribution were excluded. In addition, two taxa that were not identified as belonging to any existing taxa were described as new species. Accordingly, the genus *Porella*, which is distributed on the Korean Peninsula, was organized into 13 taxa, including 11 taxa previously recorded and 2 new-for-science taxa.

A molecular phylogenetic study was conducted on 69 specimens from 20 taxa, including 39 specimens from 13 taxa distributed on the Korean Peninsula, 28 specimens in 17 taxa known to be distributed in Northeast Asia, and 2 specimens in 1 outgroup taxa. For phylogenetic analysis, the base sequences for cpDNA *trn*L-F were determined and analyzed.

In the phylogeny tree, the cpDNA *trn*L-F region of *P. stephaniana* was revealed for the first time, and specimens from Wangpicheon Stream and Donggang River formed a single boundary group in this section.

*Porella koreana* sp. nov., described as a new species, was initially identified as *P. caespitans* based on the shape of the dorsal lobe. However, it can be distinguished from *P. caespitans*, which is 2.5–3.5 mm in plant width, has an acute apex of the dorsal lobe, and has convex trigones; the plant width is 2.5–3.8 mm; the dorsal lobe has an acuminate apex, and the trigones have a triangular shape. In the phylogenetic analysis, *P. koreana* did not form a single branch point with *P. caespitans* individuals, which supported its representation as a new taxon. Based on the characteristics of the acute apex of the dorsal lobe and the triangular or convexly thickened trigones, it was determined that it was appropriate to view it as a new taxon and was therefore described as a new species, *P. koreana* sp. nov. In addition, *P. koreana* is similar to *P. stephaniana* in that the dorsal lobe is acute; however, it can be distinguished by the absence of the tooth margin of the dorsal lobe.

*Porella japonica* individuals from Jeju Island and Japan formed one cladistic group, whereas individuals from the mainland of Woraksan Mountain, Cheongsong, Deogyusan, and Donggang formed another cladistic group. Morphological reexamination of the terrestrial *P. japonica* individuals confirmed that the bending of the lobed leaves and the small size of the central cell were distinguishing characteristics. Therefore, it was described as a new species, *P. chulii* sp. nov.

*Poella spinulosa* (Steph.) S. Hatt., which was similar to *P. gracillim* but distinguished by the presence of teeth on the ventral lobes and underleaves, formed a single cladistic group with *P. gracillim* in the ML cladistic tree. In the literature, Bakalin and Klimova [10] suggested in their study of the genus *Porella* from the Russian Far East that the morphological characteristics of *P. gracillim* were very similar to those of *P. vernicosa* and *P. spinulosa*, and further research is needed to determine the identity of *P. spinulosa*.

## 5. Materials and Methods

### 5.1. Taxon Sampling

The materials for the study were collected from various regions in Korea, China, Japan, and the Russian Far East between 2009 and 2025 and stored in the Jeonbuk National University Biology Department Herbarium (JNU), and specimens were also observed in the Hattori Botanical Garden Herbarium (NICH), Japan, and the Vladivostok Botanical Garden Herbarium (VBGI). Approximately 500 specimens collected during the study period are stored in the Jeonbuk National University Biology Department Herbarium (JNU). For phylogenetic analysis, *Ascidiota blepharophylla* ssp. *alaskana* Steere and R.M. Schust., a taxon within the genus *Ascidiota*, which is known to be closely related to the genus *Ascidiota*, was selected as an outgroup. This species is morphologically similar to the genus *Ascidiota* but differs in that the compound leaves are attached to the base of the ventral leaves in a cone shape [2,10].

### 5.2. DNA Isolation, Amplification, and Sequencing

DNA extraction was performed using specimens stored in the Jeonbuk National University Herbarium (JNU) and living organisms collected during the research period. Taxa and individuals collected as living organisms were collected and naturally dried for approximately one week. Leaves and stems were removed from the specimens with a stereomicroscope and crushed with a tissue grinder (Tissue LyserII, QIAGEN). DNA was extracted using a DNeasy Plant Mini Kit (QIAGEN, Hilden, Germany), and all processing steps followed the provided protocol ([46]; QIAGEN, Germany). DNA was extracted by mixing the extracted DNA with DNA gel loading dye (6X). After making a 1.5% agarose gel, the sample was poured; then, electrophoresis was performed. After electrophoresis, DNA extraction was confirmed using a UV transilluminator.

In this study, the noncoding region of the chloroplast DNA *trn*L-F gene was amplified with one pair of primers each, after which each nucletide sequence was analyzed.

The composition of the ploymerase chanin reaction (PCR) mixture was as follows: For *trn*L-F, FastMix Frenche PCR (iNtRON) was used. For *trn*L-F, 1.0–2.0 ng of extracted DNA and 1.0 μL of each bidirectional primer (5 pmol/μL) were mixed in a premix tube, and then distilled water was used to adjust the reaction to a total volume of 20 μL.

The primers *trn*F-C(F) and *trn*F-F(R) for the amplification of the chloroplast DNA *trn*L-F region were designed with reference to the study by Taberlet et al. [47].

*trn*L-F was predenatured at 92 °C for 120 s and then subjected to 30 thermal cycles consisting of denaturation at 95 °C for 60 s, annealing at 51 °C for 50 s, and extension at 72 °C for 90 s. After thermal cycling, a final extension step was added at 72 °C for 10 min.

The amplified PCR products were purified using a QIAquick PCR Purification Kit (QIAGEN, Germany), and all processing steps were performed according to the enclosed protocol.

### 5.3. Phylogenetic Analyses

The nucleotide sequence analysis was performed using the cycling sequencing (Biofact) method on an ABI 3730xl (Applied Biosystems Inc., Carlsbad, CA, USA) automated base sequencer. The nucleotide sequences obtained from the automated base sequencer were modified in Geneious Prime (version 2024.1, Biomatters Ltd., Auckland, New Zealand) and aligned using MUSCLE (version 5, multiple sequence comparison by log-expectation) [48]. The analyzed base sequences were also analyzed along with the data registered in GenBank (Supplementary Materials S2).

Phylogenetic analysis was performed using the maximum likelihood (ML) method. The dataset was analyzed with maximum likelihood in the program RAxML (version 8.2.11) [49] implemented in Geneious Prime (version 2024.1, Biomatters Ltd., Auckland, New Zealand). Sequence data were subjected to the GTRCAT model. A rapid bootstrap analysis with 1000 replicates and a search for the best-scoring ML tree were conducted. Nodes with bootstrap (BS) values of 70–80% were considered moderately supported, while values of 81–100% were considered well supported. The resulting tree was used to infer phylogenetic relationships.

## 6. Conclusions

Based on the integrative approach revision, the distribution of 13 species on the Korean Peninsula has been confirmed. The distribution of the other six taxa reported in the literature before our study (*P. tosana*, *P. platyphylla*, *P. perrottetiana*, *P. pinnata*, *P. spinulosa*, and *P. subobtusa*) could not be confirmed. Although the presence of at least some of them on the peninsula can be expected, there are no reliable data on their occurrence here. Two new-for-science *Porella* species have been described from the peninsula: *P. koreana* sp. nov., which is morphologically similar to *P. caespitans*, and *P. chulii* sp. nov., which somewhat resembles *P. japonica*. The segregation of both species is confirmed by molecular–genetic analyses; in addition, there are morphological features that allow for the confident identification of new taxa based only on morphological features. The status of *P. spinulosa* remains unclear, but to resolve this issue, it is necessary to study plants from the original locality (=locus classicus) where this species was described. This is especially important given some contradiction in both morphological estimations as well as the position in the phylogenetic tree of the already sequenced material available in GenBank, which, in our opinion, may be based on misidentification. To attract more attention to this genus and facilitate the work of “florist bryologists”, an identification key to all *Porella* species whose distribution on the Korean peninsula has been confirmed is provided. Each of the taxa treated is provided with descriptions and photographs compiled on the basis of a study of plants collected within Korea, which will allow, at least, for the comparison of the line-art illustrations available in the previously published literature for most species with the photographs. The list of species provided in this work is not exhaustive, and in the course of further research, some species will undoubtedly be added. Perhaps the total diversity of Porellaceae species on the Korean Peninsula may in the future approach 20 species.

## Figures and Tables

**Figure 2:**
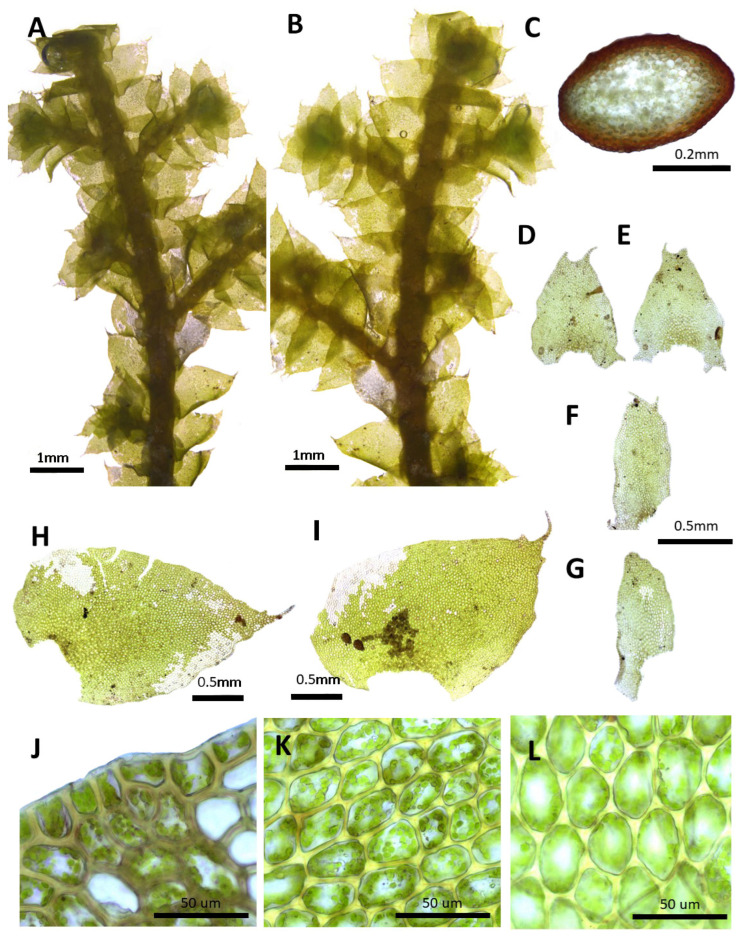
*Porella caespitans* (Steph.) S. Hatt.: (**A**) plant habit, ventral view; (**B**) plant habit, dorsal view; (**C**) cross section of the stem; (**D**,**E**) underleaves; (**F**,**G**) ventral lobes; (**H**,**I**) dorsal lobes; (**J**) marginal cells of the dorsal lobe; (**K**) middle cells of the dorsal lobe; (**L**) basal cells of the dorsal lobe. All images from Bum and S.S. Choi, 201584 (JNU).

**Table 1:** The comparison of species of the genus *Porella* on the Korean Peninsula.

Species	Apex of Dorsal Lobes	Width of Plants (mm)	Decurrent of Ventral Lobe for Stem Width	Margin of Dorsal Lobes
*P. caespitans*	Acuminate	2.5–3.8	1.0–1.2	Entire
*P. chinensis*	Obtuse and rounded	2.5–3.1	1.0–1.2	Entire with slightly crispate
*P. densifolia*	Obtuse and acute	3.0–3.75	0.1–0.2	Few teeth near the apex or entire
*P. fauriei*	Obtuse and rounded	1.2–1.5	0.1–0.2	Cilia near the apex
*P. gracillima*	Rounded and obtuse	1.5–2.0	0.1–0.2	Entire
*P. grandiloba*	Rounded and obtuse	2.5–3.1	0.1–0.2	Entire
*P. japonica*	Rounded and obtuse	1.8–2.9	0.1–0.2	Teeth or cilia near the apex
*P. oblongifolia*	Rounded and obtuse	3.0–3.5	0.2–0.3	Entire or few teeth near the apex
*P. stephaniana*	Acute	3.1–3.8	0.2–0.3	Teeth or cilia near the apex
*P. ulophylla*	Rounded and obtuse	3.2–3.8	0.2–0.3	Entire with strongly crispate
*P. vernicosa*	Obtuse and rounded	1.5–1.9	0.1–0.2	Cilia near the apex
*P. koreana*	Acute	2.5–3.5	0.3–0.5	Entire
*P. chullii*	Obtuse, rounded, and rarely acute	1.37–2.25	0.1–0.2	Entire or few teeth near the apex

## Data Availability

All data are contained within the article.

## Supplementary Materials

The following supporting information can be downloaded at https://www.mdpi.com/article/10.3390/plants14081260/s1, Supplementary Material S1. Specimens examined; Supplementary Material S2. List of materials used for DNA analysis in this study.
