# Supplementary material for: Taxonomy of the Genus Porella (Porellaceae, Marchantiophyta) on the Korean Peninsula
Source: Plants (Basel). 2025 Apr 21;14(8):1260. doi: 10.3390/plants14081260 (PMC12030336; doi:10.3390/plants14081260)
Supplement: Supplementary file 1 [file plants-14-01260-s001.zip › Supplementary Material_S1.pdf]

**SUPPLEMENTARY MATERIAL S1**

**Specimens examined**

Plants-Basel

**Taxonomy of the genus Porella (Porellaceae, Marchantiophyta) on the Korean Peninsula**

Hyun Min Bum, Seung Jin Park, Narae Yun, Vadim A. Bakalin, and Seung Se Choi

1) ***Porella caespitans*** (Steph.) S. Hatt.

Specimens examined: JJ: Jeju-si, Mt. Halla, Seongpanak-Baekrokdam, 8 Aug 2010, S.S. Choi 7712 (JNU); Seogwipo-si, Suak valley, 11 Oct 2012, S.S. Choi, 121048 (JNU). JN: Haenam-gun, Mt. Duryunsan, 18 May 2011, S.S. Choi, 110460 (JNU). GN: Hamyang-gun, Baengmudong Valley, 03 Apr 2010, S.S. Choi, 7270, (JNU); Heuksan-myeon, Is. Gageodo, 16 Jun 2018, H.M. Bum & S.S. Choi, 18120, (JNU); Hamyang-gun, Mt. Jirisan, 17 Aug 2019, H.M. Bum & S.S. Choi, 198045, 98046, 198048, 198052 (JNU); 09 Nov 2019, H.M. Bum & S.S. Choi, 1910842 (JNU); Hamyang-gun, Chilseon Valley, 10 Oct 2019, H.M. Bum & S.S. Choi, 1910128 (JNU). GB: Uljin-gun, Wangpicheon, 29 Mar 2017, H.M. Bum & S.S. Choi, 170055, 170065 (JNU); Cheongsong-gun, Windhole, 13 Oct 2015, S.J. Park, 13942 (JNU). CB: Danyang-gun, Mt. Sobaeksan, 01 Oct 2016, H.M. Bum & S.S. Choi, 161063 (JNU); 06 May 2016, H.M. Bum & S.S. Choi, 16060254 (JNU); 22 Oct 2016, H.M. Bum & S.S. Choi, 161119 (JNU); Jecheon-si, Mt. Woraksan, 05 Sep 2020, H.M. Bum & S.S. Choi, 201551, 201563, 201564, 201567, 201569, 201584 (JNU); 10 Oct 2020, H.M. Bum & S.S. Choi, 201685 (JNU); Boeun-gun, Mt. Sokrisan, 16 Nov 2019, H.M. Bum & S.S. Choi, 1910877 (JNU); 18 Oct 2019, H.M. Bum & S.S. Choi, 1910599 (JNU); 28 Oct 2019, H.M. Bum & S.S. Choi, 1910807, 1910810 (JNU). GW: Pyeongchang-gun, Mt. Odaesan, 10 Jul 2021, H.M. Bum & S.S. Choi, 210117 (JNU); Yangyang-gun, Mt. Seoraksan, 27 Sep 2020, H.M. Bum & S.S. Choi, 201645, 201651, 201652 (JNU); 29 Aug 2020, H.M. Bum & S.S. Choi, 201977, 201978 (JNU); 28 Aug 2020, H.M. Bum & S.S. Choi, 201971 (JNU); 16 Aug 2020, H.M. Bum & S.S. Choi, 201457, 201479 (JNU); 20 Jun 2020, H.M. Bum & S.S. Choi, 201308, 201309 (JNU); Yangyang-gun, Mt. Eungboksan, 15 Jan 2020, H.M. Bum & S.S. Choi, 201045, 201046 (JNU); Taebaek-si, Mt. Taebaeksan, 28 Oct 2017, H.M. Bum & S.S. Choi, 170880 (JNU); Jeongseon-gun, Windhole, 04 Oct 2015, S.J. Park, 13628 (JNU); Jeongseon-gun, Windhole, 16 Aug 2015, S.J. Park, 12748 (JNU).

2) ***Porella chinensis*** (Steph.) S. Hatt.

Specimen examined: GW: Taebaek-si, Geomnyongso, 16 Jun 2017, H.M. Bum & S.S. Choi, 170301 (JNU).

3) ***Porella densifolia*** (Steph.) S. Hatt.

Specimens examined: JN: Gwangju-si, Buk-gu, 25 Jun 2022, H.M. Bum & S.S. Choi (JNU). GB: Cheongsong-gun, Windhole, 03 Sep 2015, S.J. Park, 12872, 12891-1 (JNU).

4) ***Porella fauriei*** (Steph.) S. Hatt.,

Specimens examined: JJ: Jeju-si, Mt. Hallasan Y valley, 07 May 2017, H.M. Bum & S.S. Choi, 170180, 170181, 170197, 170252, 170255 (JNU). GN: Hamyang-gun, Mt. Jirisan, 26 Oct 2019, H.M. Bum & S.S. Choi, 1910623 (JNU). GB: Ulleung-gun, Seonginbong, 25 Sep 2021, H.M. Bum & S.S. Choi, 210277, 210294, 210298, 210311 (JNU). GW: Hongcheon-gun, Gyebangsan, 07 Aug 2021, H.M. Bum & S.S. Choi, 210155 (JNU); Yangyang-gun, Mt. Seoraksan, 14 Jun 2020, H.M. Bum & S.S. Choi, 201229 (JNU).

5) ***Porella gracillima*** Mitt.

Specimens examined: GB: Ulleung-gun, Anpyeonjeon, 25 Sep 2021, H.M. Bum & S.S. Choi, 210254 (JNU). GW: Taebaek-si, Geomnyongso, 15 Apr 2017, H.M. Bum & S.S. Choi, 170084 (JNU). Jeongseon-gun, Windhole, 04 Oct 2015, S.J. Park, 13625 (JNU); Jeongseon-gun, Donggang, 16 Aug 2010, S.S. Choi, 7913 (JNU); Samcheok-si, Mt. Deokhangsan, 14 Oct 2009, S.S. Choi, 7006b, 7020 (JNU); 20 Jul 2010, S.S. Choi, 7451, 7482 (JNU).

6) ***Porella grandiloba*** Lindb.

Specimens examined: GN: Heuksan–myeon, Is. Gageodo, 16 Jun 2018, H.M. Bum & S.S. Choi, 18107 (JNU). GB: Ulleung-gun, Seonginbong, 25 Sep 2021, H.M. Bum & S.S. Choi, 210243, 210251, 210253, 210259, 210260, 210261, 210266 (JNU); Ulleung-gun, Naribunji, 25 Sep 2021, H.M. Bum & S.S. Choi, 210242 (JNU); Ulleung-gun, Anpyeonjeon, 25 Sep 2021, H.M. Bum & S.S. Choi, 210258 (JNU); Cheongsong-gun, Windhole, 03 Sep 2015, S.J. Park, 12874 (JNU). CB: Jecheon-si, Mt. Woraksan, 05 Sep 2020, H.M. Bum & S.S. Choi, 201570 (JNU); Danyang-gun, Mt. Sobaeksan, 01

Oct 2016, H.M. Bum & S.S. Choi, 161072, 161080 (JNU); 06 Aug 2016, H.M. Bum & S.S. Choi, 160804 (JNU); Boeun-gun, Bukdumun Windhole, 30 Sep 2015, S.J. Park, 13326, 13344, 13347, 13353, 13358, 13369, 13374 (JNU); Boeun-gun, Bukdumun Windhole, 16 Jul 2014, S.J. Park, 10878, 10889 (JNU). GW: Yangyang-gun, Mt. Seoraksan, 27 Sep 2020, H.M. Bum & S.S. Choi, 201647 (JNU); 16 Aug 2020, H.M. Bum & S.S. Choi, 201467 (JNU); 14 Jun 2020, H.M. Bum & S.S. Choi, 201298 (JNU); 13 Jun 2020, H.M. Bum & S.S. Choi, 201268 (JNU); Inje-gun, Mt. Galjeongokbong, 15 Jan 2020, H.M. Bum & S.S. Choi, 201028 (JNU); Taebaek-si, Mt. Taebaeksan, 28 Oct 2017, H.M. Bum & S.S. Choi, 170868, 170869, 170873, 170874 (JNU); 16 Sep 2017, H.M. Bum & S.S. Choi, 170603 (JNU); Taebaek-si, Geomnyongso, 16 Jun 2017, H.M. Bum & S.S. Choi, 170300 (JNU); 15 Apr 2017, H.M. Bum & S.S. Choi, 170086 (JNU); 05 Apr 2017, H.M. Bum & S.S. Choi, 170089 (JNU); 04 Oct 2015, S.J. Park, 21509 (JNU); Hongcheon-gun, Windhole, 23 Sep 2015, S.J. Park, 13227, 13261, 13301 (JNU).

7) ***Porella japonica*** (Sande Lac.) Mitt.,

Specimens examined: JJ: Jeju-si, Sangdeokcheon, 10 Jan 2022, H.M. Bum & S.S. Choi, 120342 (JNU); Jeju-si, Manjang cave, 19 Jan 2020, H.M. Bum & S.S. Choi, 201080a (JNU); Seogwipo-si, Suak valley, 11 Oct 2012, S.S. Choi, 121036, 121052 (JNU); Jeju-si, Musucheon, 18 Mar 2012, S.S. Choi, 120103, 120104, 120138 (JNU); Seogwipo-si, Hyodoncheon, 30 Oct 2011, S.S. Choi, 111328 (JNU); 01 Mar 2012, S.S. Choi, 120416 (JNU); ; Seogwipo-si, Seondol valley, 20 Jun 2011, S.S. Choi, 110821 (JNU); 18 Sep 2011, S.S. Choi, 110931 (JNU).

8) ***Porella oblongifolia*** S. Hatt.,

Specimens examined: JB; Muju-gun, Mt. Deogyusan, 27 Jun 2008, S.S. Choi, 843 (JNU). CB: Jecheon-si, Mt. Woraksan, 05 Sep 2020, H.M. Bum & S.S. Choi, 201558, 201570, 201574, (JNU). GW: Yangyang-gun, Mt. Eungboksan, 15 Jan 2020, H.M. Bum & S.S. Choi, 201043 (JNU); Goseong-gun, Mt. Masanbong, 08 May 2019, H.M. Bum & S.S. Choi, 19073 (JNU); Taebaek-si, Mt. Taebaeksan, 28 Oct 2017, H.M. Bum & S.S. Choi, 170870 (JNU).

9) ***Porella stephaniana*** (C. Massal.) S. Hatt.,

Specimens examined: GB: Uljin-gun, Wangpicheon, 29 Mar 2017, H.M. Bum & S.S. Choi, 170067 (JNU). GW: Taebaek-si, Geomnyongso, 15 Apr 2017, H.M. Bum & S.S. Choi, 170093, 170094 (JNU); Jeongseon-gun, Donggang, 16 Aug 2010, S.S. Choi, 7920 (JNU); 17 Aug 2010, S.S. Choi, 7937, 7938 (JNU).

10) ***Porella ulophylla*** (Steph.) S. Hatt.,

Specimens examined: JJ: Jeju-si, Mt. Halla, Seongpanak-Baekrokdam, 8 Aug 2010, S.S. Choi 7708 (JNU); Jeju-si, Che Oruem, 27 Aug 2010, S.S. Choi 8068 (JNU); Jeju-si, Musu stream, Goangryeong 2nd Bridge, 17 Mar 2012, S.S. Choi 120076 (JNU); 17 Mar 2012, S.S. Choi 120065 (JNU); Jeju-si, Dongbaekdongsan, 22 Mar 2012, S.S. Choi 120269, 120271, 120278 (JNU); 16 Oct 2012, S.S. Choi 121140 (JNU); Jeju-si, Tamla valley, 10 Apr 2012, S.S. Choi 120317 (JNU); Jeju-si, Gwangryeongcheon stream, 4 May 2012, S.S. Choi 120491 (JNU); Seogwipo-si, Hyodon stream, 7 Aug 2010, S.S. Choi 7703 (JNU); Seogwipo-si, Hannam forest, 3 Nov 2011, S.S. Choi 111497 (JNU); Seogwipo-si, Mt. Hanla, Witse Oreum, 2 May 2012, S.S. Choi 120433 (JNU); Seogwipo-si, Bolrae Oreum, 5 Sep 2012, S.S. Choi 120725 (JNU); Seogwipo-si, Seondol valley, 18 Sep 2011, S.S. Choi 110941 (JNU). JN: Is. Gageodo, Heuksan-myeon, 16 Jun 2018, H.M. Bum & S.S. Choi, 18104 (JNU); Goheung-gun, Mt. Palyeoung, ridge, 23 Jun 2009, S.S. Choi 4035 (JNU); Goheung-gun, Is. Oenarodo, Mt. Bongrae, valley, 20 May 2011, S.S. Choi 110594 (JNU); Gurye-gun, Mt. Jiri, Banyabong, 5 Aug 2010, S.S. Choi 7563 (JNU); Jangheung-gun, Mt. Suin, valley, 24 Jun 2009, S.S. Choi 4046 (JNU); Jindo-gun, Mt. Yegwi, ridge, 11 Feb 2010, S.S. Choi 7106 (JNU); Sinan-gun, Is. Gageo, The 2nd villiate-Lighthouse, 2 Mar 2010, S.S. Choi 7208 (JNU); Sinan-gun, Is. Gageo, Lighthouse-Mt. Doksil, 2 Mar 2010, S.S. Choi 7220 (JNU); Wando-gun, Sangwhangbong, valley, 9 Feb 2010, S.S. Choi 3209, 7018 (JNU); . JB: Buan-gun, Mt. Naebyen, Beadrock near road, 10 Mar 2009, S.S. Choi 3389 (JNU); Jeongeup-si, Mt. Naejang, Geumseon valley, 16 Mar 2009, S.S. Choi 3498 (JNU); Muju-gun, Mt. Jeoksang, below Waterfall, 22 Mar 2009, S.S. Choi 3409 (JNU); Namwon-gun, Mt. Jiri,

Forest lodge, 28 May 2011, S.S. Choi 110666 (JNU); Namwon-si, Mt. Jiri, Hansin valley, 7 Oct 2009, S.S. Choi 6032, 6035 (JNU). GN: Hamyang-gun, Mt. Jirisan, 17 Aug 2019, H.M. Bum & S.S. Choi, 198042, 198060, 198062 (JNU); Miryang-si, Windhole, 14 Oct 2015, S.J. Park, 14416-1, 14442 (JNU); Hapcheon-gun, Mt, Gaya, Baekwondong area, 8 Sep 2009, S.S. Choi 4339 (JNU); Sancheong-gun, Mt. Jiri, Jangteomok shelter, 16 Jun 2009, S.S. Choi 3840 (JNU); 16 Jun 2009, S.S. Choi 3846 (JNU); Tongyeong-si, Mt. Mireuk, stony field, 17 Mar 2011, S.S. Choi 110058 (JNU). GB: Uljin-gun, Mt. Chilbosan, 27 Mar 2017, H.M. Bum & S.S. Choi, 170025 (JNU); Cheongsong-gun, Windhole, 13 Oct 2015, S.J. Park, 13877, 13891, 13902, 13910 (JNU); Uiseong-gun, Windhole, 12 Oct 2015, S.J. Park, 13859 (JNU). CB: Yeongdong-gun, Mt. Minjujisan, Forest lodge, 19 May 2012, S.S. Choi 120589 (JNU); Jecheon-si, Mt. Woraksan, 22 Aug 2020, H.M. Bum & S.S. Choi, 201496 (JNU); Boeun-gun, Mt. Sokrisan, 16 Nov 2019, H.M. Bum & S.S. Choi, 1910876 (JNU); Danyang-gun, Mt. Sobaeksan, 01 Oct 2016, H.M. Bum & S.S. Choi, 161065 (JNU); 10 Sep 2016, H.M. Bum & S.S. Choi, 161040 (JNU); Boeun-gun, Bukdumun Windhole, 30 Sep 2015, S.J. Park, 13356, 13362, 13376 (JNU); 16 Jul 2014, S.J. Park, 10882 (JNU). GW: Inje-gun, Mt. Seolak, Hangyeoryeong, 21 Sep 2009, S.S. Choi 5053 (JNU); Samcheok-si, Mt. Deokhang, valley, 20 Jul 2010, S.S. Choi 7460 (JNU); Sokcho-si, Mt. Seolak, Biseondae, 11 Oct 2010, S.S. Choi 8340 (JNU); Wonju-si, Mt. Chiak, Seryeom waterfall, 31 Jul 2009, S.S. Choi 4155, 4164 (JNU); Pyeongchang-gun, Mt. Odaesan, 30 May 2021, H.M. Bum & S.S. Choi, 210014, 201544, 201545 (JNU); Yangyang-gun, Mt. Seoraksan, 14 Jun 2020, H.M. Bum & S.S. Choi, 201299 (JNU); 16 May 2020, H.M. Bum & S.S. Choi, 201228 (JNU); Taebaek-si, Mt. Taebaeksan, 28 Oct 2017, H.M. Bum & S.S. Choi, 170896 (JNU); Jeongseon-gun, Windhole, 16 Aug 2015, S.J. Park, 12749 (JNU).

11) ***Porella vernicosa*** Lindb.

Specimens examined: JJ: Jeju-si, Tamla valley, 10 Nov 2018, H.M. Bum & S.S. Choi, 180321 (JNU); Jeju-si, Jeju Stone Park, 13 Aug 2017, H.M. Bum & S.S. Choi, 170527, 170528, 170529 (JNU); Jeju-si, Gotjawal, 30 Jun 2017, H.M. Bum & S.S. Choi, 170422, 170430 (JNU); Jeju-si, Musucheon, 17 Mar 2012, S.S. Choi, 120076 (JNU); Seogwipo-si, Seondol valley, 18 Sep 2011, S.S. Choi, 110918 (JNU). JN: Goheung-gun, Is. Oenarodo, 20 Mar 2011, S.S. Choi, 110584 (JNU). JB; Jinan-gun, Windhole, 08 Oct 2015, S.J. Park, 13760 (JNU). GN: Hamyang-gun, Mt. Jirisan, 23 Dec 2021, H.M. Bum & S.S. Choi, B21357 (JNU); 09 Nov 2019, H.M. Bum & S.S. Choi, 1910840, 1910841 (JNU); 26 Oct 2019, H.M. Bum & S.S. Choi, 1910641 (JNU); 17 Aug 2019, H.M. Bum & S.S. Choi, 198047, 198049, 198050, 198051, 198053 (JNU). GB: Ulleung-gun, Seonginbong, 25 Sep 2021, H.M. Bum & S.S. Choi, 210234, 210263 (JNU); Cheongsong-gun, Windhole, 13 Oct 2015, S.J. Park, 13915-1 (JNU); 03 Sep 2015, S.J. Park, 12883, 12901 (JNU); Uiseong-gun, Windhole, 12 Oct 2015, S.J. Park, 13844, 13846 (JNU). CB: Jecheon-si, Mt. Woraksan, 05 Sep 2020, H.M. Bum & S.S. Choi, 201559 (JNU); 22 Aug 2020, H.M. Bum & S.S. Choi, 201502 (JNU); 01 Aug 2020, H.M. Bum & S.S. Choi, 201441 (JNU); 30 May 2020, H.M. Bum & S.S. Choi, 201254 (JNU); 01 May 2020, H.M. Bum & S.S. Choi, 201448 (JNU); 25 Apr 2020, H.M. Bum & S.S. Choi, 201164 (JNU); Boeun-gun, Mt. Sokrisan, 16 Nov 2019, H.M. Bum & S.S. Choi, 1910871, 1910875 (JNU); 28 Oct 2019, H.M. Bum & S.S. Choi, 1910806, 1910808 1910809, (JNU); 18 Oct 2019, H.M. Bum & S.S. Choi, 1910547, 1910601 (JNU); Danyang-gun, Mt. Sobaeksan, 01 Oct 2016, H.M. Bum & S.S. Choi, 161062 (JNU); 30 Sep 2016, H.M. Bum & S.S. Choi, 161060 (JNU); 10 Sep 2016, H.M. Bum & S.S. Choi, 161043 (JNU); Boeun-gun, Bukdumun Windhole, 30 Sep 2015, S.J. Park, 13342,13351 (JNU). GW: Pyeongchang-gun, Mt. Odaesan, 11 Sep 2021, H.M. Bum & S.S. Choi, 210194, 210196 (JNU); 10 Jul 2021, H.M. Bum & S.S. Choi, 210118 (JNU); 21 Jun 2021, H.M. Bum & S.S. Choi, 210042 (JNU); 30 May 2021, H.M. Bum & S.S. Choi, 210015, 210021, 210024 (JNU); Yangyang-gun, Mt. Seoraksan, 27 Sep 2020, H.M. Bum & S.S. Choi, 201642, 201643, 201650 (JNU); 29 Aug 2020, H.M. Bum & S.S. Choi, 20128, 201522, 201969 (JNU); 16 Aug 2020, H.M. Bum & S.S. Choi, 201458 (JNU); 20 Jun 2020, H.M. Bum & S.S. Choi, 201305 (JNU); Inje-gun, Mt. Galjeongokbong, 15 Jan 2020, H.M. Bum & S.S. Choi, 201036 (JNU); Yangyang-gun, Mt. Eungboksan, 15 Jan 2020, H.M. Bum & S.S. Choi, 201044 (JNU); Taebaek-si, Mt. Taebaeksan, 16 Sep 2017, H.M. Bum & S.S. Choi, 170598 (JNU); 15 Apr 2017, H.M. Bum & S.S. Choi, 170092 (JNU); Taebaek-si, Geomnyongso, 16 Jun 2017, H.M. Bum & S.S. Choi, 170305 (JNU); Jeongseon-gun, Windhole, 16 Aug 2015, S.J. Park, 12737-1 (JNU).

12) ***Porella koreana*** H.M. Bum, S.J. Park, Bakalin & S.S. Choi

Specimens examined: GB: Cheongsong-gun, wind-hall, 13 Oct 2015, S.J. Park, 13899 (HIBR); HNIBRMS3108 (HIBR).

13) ***Porella chulii*** H.M. Bum, S.J. Park, Bakalin & S.S. Choi

Specimens examined: JB: Muju-gun, Mt. Deogyusan, 24 Jun 2009, S.S. Choi, 4048 (JNU). CB: Jecheon-si, Mt. Woraksan, 05 Sep 2020, H.M. Bum & S.S. Choi, 201573 (JNU); Cheongsong-gun, Windhole, 16 Jul 2014, S.J. Park, 10899-2 (JNU). GW: Taebaek-si, Mt. Taebaeksan, Gyeomryoungso, 15 Apr 2017, H.M. Bum & S.S. Choi, 170090 (HIBR); HNIBRMS6, (HIBR)
