# Supplementary material for: Taxonomy of the Genus Porella (Porellaceae, Marchantiophyta) on the Korean Peninsula
Source: Plants (Basel). 2025 Apr 21;14(8):1260. doi: 10.3390/plants14081260 (PMC12030336; doi:10.3390/plants14081260)
Supplement: Supplementary file 1 [file plants-14-01260-s001.zip › Supplementary Material_S2.pdf]

SUPPLEMENTARY MATERIAL S2

List of materials used for DNA analysis in this study

Plants-Basel

Taxonomy of the genus Porella (Porellaceae, Marchantiophyta) on the Korean Peninsula

Hyun Min Bum, Seung Jin Park, Narae Yun, Vadim A. Bakalin, and Seung Se Choi

Table. Vouchers and GenBank accession numbers for taxa used in the molecular phylogenetic analysis. Taxon name, locality, Voucher (collector, number), and Genbank accession number for DNA sequences *trn*L-F. Sequences marked with an asterisk (*) were newly obtained in this study.

Species	Locality	Voucher	Accession
Porella acutifolia	Indonesia		EF545404
	Papua New Guinea		EF545405
Porella caespitans	North Korea		EF545412
	China		EF545413
	China		EF545409
	South Korea: Chungcheongbuk-do, Woraksan	H.M. Bum & S.S. Choi 201584	PV543506*
	South Korea: Gyeongsangnam-do, Jirisan	H.M. Bum & S.S. Choi 198046	PV543505*
	South Korea: Jeollanam-do, Gageodo	H.M. Bum & S.S. Choi 18120	PV543504*
Porella chinensis	Russia		EF545407
	South Korea: Gangwon-do, Taebaeksan	H.M. Bum & S.S. Choi 170301	PV543507*
Porella chulii	South Korea: Chungcheongbuk-do, Woraksan	H.M. Bum & S.S. Choi 201573	PV543511*
	South Korea: Chungcheongbuk-do, Cheong-gun	S.J. Park 108992	PV543509*
	South Korea: Jeollabuk-do, Deogyusan	H.M. Bum & S.S. Choi 4048	PV543508*
	South Korea: Gangwon-do, Donggang	H.M. Bum & S.S. Choi 170090	PV543510*
Porella densifolia	Japan		EF545440
	Japan		EF545439
	China		EF545435
	South Korea: Gyeongsangbuk-do, Cheongsong	S.J. Park 13882	PV543512*
Porella fauriei	Japan		EF545416
	South Korea: Gangwon-do, Gyebangsan	S.J. Park 210155	PV543514*
	South Korea: Gyeongsangbuk-do, Ulleung	H.M. Bum & S.S. Choi 210277	PV543515*
	South Korea: Gangwon-do, Seoraksan	H.M. Bum & S.S. Choi 201229	PV543513*
Porella gracillima	China		EF545450
	Russia		EF545451
	South Korea: Gyeongsangbuk-do, Ulleung	H.M. Bum & S.S. Choi 210254	PV543517*
	South Korea: Gangwon-do, Donggang	S.S. Choi 7913	PV543516*
Porella grandiloba	Japan		EF545486
	North Korea		EF545395
	South Korea: Chungcheongbuk-do, Woraksan	H.M. Bum & S.S. Choi 201568	PV543520*
	South Korea: Chungcheongbuk-do, Woraksan	H.M. Bum & S.S. Choi 201570	PV543521*

	South Korea: Gyeongsangbuk-do, Ulleung	H.M. Bum & S.S. Choi 210242	PV543524*
	South Korea: Chungcheongbuk-do, Woraksan	H.M. Bum & S.S. Choi 201166	PV543518*
	South Korea: Chungcheongbuk-do, Woraksan	S.S. Choi 201575	PV543522*
	South Korea: Gyeongsangbuk-do, Ulleung	H.M. Bum & S.S. Choi 210258	PV543525*
	South Korea: Gangwon-do, Odaesan	H.M. Bum & S.S. Choi 201644	PV543523*
	South Korea: Chungcheongbuk-do, Woraksan	S.S. Choi 201500	PV543519*
Porella japonica	Japan		EF545396
	South Korea: Jeju-do, Jeju	S.S. Choi 201080a	PV543527*
	South Korea: Jeju-do, Jeju	H.M. Bum & S.S. Choi 120104	PV543526*
Porella koreana	South Korea: Gyeongsangbuk-do, Cheongsong	H.M. Bum & S.S. Choi 13899	PV543528*
Porella oblongifolia	Bhutan		EF545438
	South Korea: Gangwon-do, Odaesan	H.M. Bum & S.S. Choi 210114	PV543532*
	South Korea: Gangwon-do, Taebaeksan	H.M. Bum & S.S. Choi 170870	PV543529*
	South Korea: Chungcheongbuk-do, Woraksan	H.M. Bum & S.S. Choi 201558	PV543531*
	South Korea: Gangwon-do, Eungboksan	H.M. Bum & S.S. Choi 201043	PV543530*
Porella perrottetiana	Bhutan		EF545401
	Japan		EF545401
Porella platyphylla	Germany		EF545384
	Bulgaria		EF545385
	Italy		EF545386
Porella spinulosa	Japan		EF545452
Porella stephaniana	South Korea: Gangwon-do, Wangpicheon	H.M. Bum & S.S. Choi 170067	PV543533*
	South Korea: Gangwon-do, Donggang	H.M. Bum & S.S. Choi 170093	PV543534*
Porella subobtusa	Japan		EF545411
Porella ulophylla	China		EF545408
	South Korea: Gyeongsangbuk-do, Cheongsong	H.M. Bum & S.S. Choi 13891	PV543535*
	South Korea: Gangwon-do, Odaesan	H.M. Bum & S.S. Choi 210014	PV543537*
Porella ulophylla	South Korea: Gangwon-do, Seoraksan	S.J. Park 201545	PV543536*
Porella vernicosa	North Korea		EF545454
	Russia		EF545456
	North Korea		EF545455
	South Korea: Jeju-do, Jeju	S.J. Park 170527	PV543538*
	South Korea: Gangwon-do, Seoraksan	H.M. Bum & S.S. Choi 201643	PV543540*
	South Korea: Gyeongsangnam-do, Jirisan	H.M. Bum & S.S. Choi B21357	PV543542*

	South Korea: Gyeongsangbuk-do, Ulleung	H.M. Bum & S.S. Choi 210234	PV543541*
	South Korea: Chungcheongbuk-do, Woraksan	H.M. Bum & S.S. Choi 201448	PV543539*
Ascidiota blepharophylla ssp. Alaskana	USA: Alaska	(outgroup)	EF545376
